# Computational and experimental insights into the interaction of the seaweed-derived steroidal metabolite 11α-hydroxyprogesterone with the glucocorticoid receptor

**DOI:** 10.1016/j.csbj.2025.12.028

**Published:** 2025-12-30

**Authors:** Supatchar Sermsakulwat, Phuphiphat Jaikaew, Tiwtawat Napiroon, Worawat Surarit, Thrissawan Traijitt, Theppanya Charoenrat, Bodee Nutho, Arachaporn Thong-olran, Supenya Chittapun

**Affiliations:** a Department of Biotechnology, Faculty of Science and Technology, Thammasat University, Rangsit Center, Pathum Thani 12121, Thailand; b Department of Pharmacology, Faculty of Science, Mahidol University, Ratchathewi, Bangkok 10400, Thailand

**Keywords:** Seaweed metabolites, Glucocorticoid receptor, Molecular docking, Molecular dynamic, Anti-inflammatory activity, ADMET prediction

## Abstract

Seaweed-derived metabolites offer a rich source of bioactive compounds with therapeutic potential. In this study, 112 steroidal metabolites from *Sargassum polycystum*, *Gracilaria fisheri*, and *Caulerpa lentillifera* were computationally screened to identify candidates interacting with the glucocorticoid receptor (GR), a key molecule regulating inflammatory signaling. Among them, 11α-hydroxyprogesterone (SW052) from *S. polycystum* exhibited the favorable predicted GR binding affinity (−11.6 kcal/mol), comparable to hydrocortisone and medrysone. Molecular docking and 500 ns molecular dynamics simulations revealed a stable GR-SW052 complex stabilized by hydrophobic and van der Waals interactions with MET560, LEU563, LEU566, MET601, MET604, LEU608, LEU732, TYR735, and CYS736. Free energy analysis (MM/PBSA) supported favorable thermodynamic binding, and *in silico* ADMET evaluation predicted good oral absorption and low toxicity. *In vitro* assays showed that both SW052 and seaweed lipophilic extract were non-cytotoxic (>70 % cell viability) and significantly inhibited nitric oxide (NO) production in lipopolysaccharide-stimulated RAW 264.7 macrophages (IC_50_ = 144.05 ± 8.06 and 108.24 ± 4.64 µg/mL, respectively). This study establishes an integrated computational-experimental framework for prioritizing seaweed-derived steroidal metabolites targeting the GR. Using this framework, SW052 was identified as a potential natural compound with stable predicted GR engagement, favorable *in silico* pharmacokinetic properties, and NO suppression, providing a structure-guided basis for further mechanism and *in vivo* validation.

## Highlights

• Integrated computational-experimental workflow was applied to prioritize seaweed-derived steroidal metabolites targeting GR.
• The seaweed-derived steroidal metabolite 11α-hydroxyprogesterone shows favorable predicted binding to GR.
• Molecular dynamics simulations demonstrate a structurally stable GR-SW052 complex over 500ns.
• SW052 exhibits docking-based binding affinity comparable to reference glucocorticoids used clinically.
• *In vitro* assays indicate non-cytotoxicity and dose-dependent inhibition of nitric oxide production.

## Graphical Abstract

**Figure:**
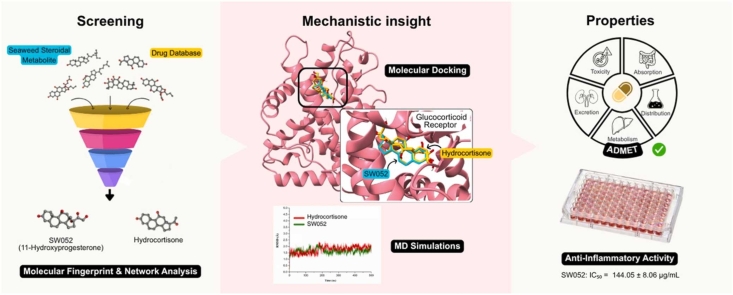

## 1 Introduction

Inflammation is a fundamental biological response that protects the host from pathogens, toxins, and injury. It involves vascular alterations, immune cell infiltration, and the release of cytokines, prostaglandins, and reactive oxygen species (ROS), which together coordinate pathogen clearance and tissue repair [1], [2]. Acute inflammation is advantageous, however, its prolonged presence leads to chronic ailments, including arthritis, cardiovascular disorders, metabolic syndrome, and cancer [3]. Present anti-inflammatory medications, such as non-steroidal anti-inflammatory drugs (NSAIDs) and corticosteroids, are efficacious yet constrained by side effects. NSAIDs can induce gastrointestinal complications due to suppression of cytoprotective prostaglandins [4], [5], elevate cardiovascular risk through altered thrombotic balance [6], [7], and impair renal function [8]. Prolonged corticosteroid therapy induces metabolic dysfunction, osteoporosis, and immune suppression [9]. These safety issues show how important it is to find new, safer, and more long-lasting anti-inflammatory drugs.

Marine macroalgae (seaweeds) have emerged as prolific sources of structurally diverse natural products with pharmaceutical potential. Brown, red, and green algae produce metabolites such as polysaccharides, phlorotannins, carotenoids, peptides, polyunsaturated fatty acids, and sterols that exhibit antioxidant, immunomodulatory, antiviral, antidiabetic, anticancer, and anti-inflammatory properties [10], [11], [12]. For example, phlorotannins reduce oxidative stress and inflammation while enhancing glucose metabolism [13], [14], [15]. Sulfated polysaccharides such as fucoidan modulate immune function and improve gut health [16], [17]. The carotenoid fucoxanthin contributes to anti-obesity and photoprotective effects [18], [19]. Seaweed-derived sterols like fucosterol demonstrate anticancer [20], anti-inflammatory [21], and cardioprotective activities [22], [23]. Collectively, this chemical richness suggests that macroalgal metabolites may act on diverse molecular pathways, positioning them as promising scaffolds for next-generation pharmaceuticals and nutraceuticals [11], [12], [24], [25]. Nevertheless, despite the breadth of evidence, the precise molecular targets, structure–function relationships, and therapeutic mechanisms of many seaweed-derived metabolites remain poorly defined [10], [26].

Steroidal compounds are particularly significant among seaweed metabolites due to their structural resemblance to endogenous hormones and their potential to modulate nuclear receptors. The glucocorticoid receptor (GR) is a ligand-activated transcription factor and a key biological macromolecule that regulates immune and inflammatory responses by suppressing cytokine production, inhibiting pro-inflammatory enzymes, and modulating immune cell activity [27], [28]. Synthetic glucocorticoids remain the standard therapy for inflammatory diseases, however, their long-term use frequently causes metabolic imbalance, osteoporosis, and immunosuppression [29]. In contrast, natural steroidal metabolites from marine algae, such as fucosterol, have demonstrated bioactivities, including anti-inflammatory and antioxidant effects, with potentially improved safety and biocompatibility [20]. Nevertheless, many seaweed-derived steroidal metabolites remain unexplored in terms of their structural and pharmacophoric similarity to corticosteroids and their potential to serve as bio-sustainable anti-inflammatory candidates. A systematic evaluation of their similarity to clinically used anti-inflammatory drugs, combined with *in silico* pharmacokinetic evaluation and preliminary cell-based cytotoxicity and nitric oxide inhibition assessment, represents a green drug discovery strategy for prioritizing promising marine-derived steroidal candidates. Such an approach can accelerate early-stage lead identification while reducing experimental cost, limiting animal use, and supporting the sustainable development of marine-derived anti-inflammatory agents.

To address this gap, the present study aimed to systematically identify and preliminarily evaluate seaweed-derived steroidal metabolites with predicted relevance to GR binding through an integrative computational-experimental approach. A curated library of 112 metabolites from *Sargassum polycystum*, *Gracilaria fisheri*, and *Caulerpa lentillifera* was assembled and analyzed using cheminformatics-based similarity mapping, network pharmacology, and protein–protein interaction analyses to prioritize candidate steroidal compounds. Molecular docking and molecular dynamics (MD) simulations were then employed to characterize the predicted structural basis of GR–ligand interactions, while *in silico* ADMET predictions provided insight into pharmacokinetic and safety profiles. Finally, the prioritized candidate, 11α-hydroxyprogesterone (SW052) and the lipophilic extract, were subjected to preliminary *in vitro* cytotoxicity and nitric oxide inhibition assays in lipopolysaccharide (LPS)-stimulated macrophages. This multidisciplinary workflow integrates cheminformatics, molecular simulation, and cell-based screening to provide structure-guided insight into the potential relevance of seaweed-derived steroidal metabolites to the GR and to facilitate their prioritization in sustainable, green drug discovery pipelines.

## 2 Materials and methods

### 2.1 Metabolite library construction

A curated set of 112 secondary metabolites was compiled from three seaweeds, including *Sargassum polycystum*, *Gracilaria fisheri*, and *Caulerpa lentillifera*, as reported by Thong-olran et al. [30]. In short, dried algal biomass was macerated in methanol and then separated into lipophilic and hydrophilic fractions. Metabolites in the lipophilic extract were analyzed using GC–MS, and compound identification was confirmed by comparison with the NIST MS Search Program (Version 2.0), yielding match factor scores of 700–800. In the present work, the chemical structures of these metabolites were verified and standardized against major repositories, including the Seaweed Metabolite Database (https://www.swmd.co.in/) [31], PubChem (https://pubchem.ncbi.nlm.nih.gov/) [32], ChEMBL (https://www.ebi.ac.uk/chembl/) [33], SpectraBase (https://spectrabase.com/) and ChemSpider (https://www.chemspider.com/) [34], all databases were downloaded in September 2024 and exported as SMILES (Simplified Molecular Input Line Entry System).

Approved drugs were retrieved from DrugBank version 5.1.10 (downloaded January 2023 from https://go.drugbank.com/) [35]. The XML file was parsed in Python 3.12.7 (CPython) using Anaconda3 (Navigator 2.6.3) and Spyder 5.5.1 on Windows with custom scripts based on regular expression pattern matching following Han et al. [36]. Only drug entries with valid SMILES were retained, while duplicates or records lacking structural information were excluded.

### 2.2 Fingerprint-based molecular similarity screening

Structural similarity between seaweed-derived metabolites and approved drugs was assessed using the RDKit cheminformatics library, version 2023.03.2 in Python 3.12.7 (Anaconda3; Spyder 5.5.1). SMILES were transformed into multiple molecular fingerprints to capture diverse structural features, including Daylight-like path fingerprints (1024 bits) [37], MACCS keys (166 predefined features) [38], circular Morgan fingerprints (radius = 2, 2048 bits), and Extended-Connectivity Fingerprints (ECFP6, radius = 3; 2048 bits) [39]. Pairwise similarities were quantified using the Tanimoto coefficient (Tc) [40]. A similarity threshold of ≥ 0.85, as recommended in cheminformatics benchmarking studies [41], was applied to identify high-confidence structural analogs. This strong threshold was intentionally selected to ensure scaffold-level similarity, particularly for rigid steroidal frameworks, where minor structural variations can lead to substantial differences in receptor binding, metabolism, and biological activity. Metabolite–drug pairs were retained using a union-based strategy, whereby a metabolite was considered structurally similar if it met the similarity threshold in at least one fingerprint method. A metabolite meeting this criterion with at least one approved drug was retained for subsequent pharmacological classification.

### 2.3 Therapeutic classification of structurally similar drugs

Drugs with structural similarity scores of ≥ 0.85 (Tc) to seaweed-derived metabolites were selected for therapeutic annotation. Metadata including DrugBank identifiers, generic names, molecular formulas, SMILES, descriptions, pharmacodynamic profiles, and therapeutic indications were extracted from the DrugBank XML dataset [42] using keyword-based parsing in Python 3.12.7 (Anaconda3; Spyder 5.5.1). Associated protein targets and their UniProt accession numbers were retrieved in parallel from UniProt [43]. The classification process was guided by keyword mapping and functional annotations, following the chemical and pharmacological curation methods described by Dunkel et al. [41]. Selected drugs were categorized into 14 therapeutic classes based on the World Health Organization's Anatomical Therapeutic Chemical (ATC) classification system (https://www.who.int/tools/atc-ddd-toolkit/atc-classification) [44], using previously extracted metadata and functional keyword mapping; ATC labels were assigned at level 1 (14 anatomical/pharmacological main classes) and level 2 (pharmacological/therapeutic subclasses). To capture anti-inflammatory relevance beyond ATC classes, an additional functional-keyword classification (e.g., “inflammation", “inflammatory", and “anti-inflammatory”) was applied to prioritize compounds. This classification covered all drug types and statuses, including prodrugs and withdrawn products.

### 2.4 Identification of inflammation-related protein targets

Inflammation-associated protein targets were identified through systematic screening of DisGeNET (http://www.disgenet.org/) [45] and UniProt (http://www.uniprot.org/) [43]. Keyword-based searches using the terms “inflammation” and “inflammatory” were performed in November 2024, restricted to *Homo sapiens*. Databases retrieved from each database were cross-compared, and intersecting entries were considered high-confidence targets with robust annotation across independent resources. Venn diagram analysis was performed using the VENNY2.1 tool (https://bioinfogp.cnb.csic.es/tools/venny/) to visualize overlapping proteins. The intersected set of proteins was subsequently selected for network-based analyses.

### 2.5 Network-based analysis of inflammation-related proteins

#### 2.5.1 Protein-protein interaction (PPI) network

To investigate the molecular interconnectivity among inflammation-associated targets, a protein–protein interaction (PPI) network was constructed using the STRING version 12.0 (https://string-db.org) [46], restricted to *H. sapiens*. A minimum interaction confidence score of 0.7 was applied to ensure high-confidence associations, following STRING recommendations and prior studies. Interaction data were exported in TSV format and visualized in Cytoscape version 3.10.2 [47]. Network topology was analyzed using the Network Analyzer tool, and centrality metrics, including degree centrality (DC), closeness centrality (CC), and betweenness centrality (BC) [48], were calculated. Proteins exhibiting high centrality were retained for docking and network integration analyses to identify potential regulatory mediators of inflammatory signaling.

#### 2.5.2 Metabolite–drug–target interaction network

To integrate chemical and biological data, a metabolite-drug-target interaction network was constructed in Cytoscape version 3.10.2. The network incorporated three entities: (i) seaweed-derived bioactive metabolites, (ii) structurally similar DrugBank pharmaceuticals (Tc ≥ 0.85), and (iii) inflammation-related proteins identified from curated databases. Structural similarity and drug target associations guided the inference of metabolite-target relationships [41]. Networks were merged using Cytoscape version 3.10.2 Merge Networks function, with nodes annotated by entity type and visually distinguished by color. Node size was scaled according to degree centrality to emphasize highly connected elements. Additional refinements and consistency checks were conducted with customized Python 3.12.7 (Anaconda3; Spyder 5.5.1). This integrated network provided a systems-level perspective of chemical–biological interactions and enabled prioritization of seaweed-derived metabolites with putative anti-inflammatory relevance.

### 2.6 Molecular docking of inflammation-related proteins

Molecular docking simulations were performed to evaluate the binding affinities and interaction profiles of seaweed-derived metabolites and structurally analogous anti-inflammatory drugs against inflammation-associated protein targets. This approach aimed to predict molecular compatibility interactions at the atomic level.

#### 2.6.1 Inflammation-related proteins and seaweed metabolite prioritization for molecular docking

Inflammation-related protein and seaweed metabolite selection was performed using a multistep prioritization workflow. Initially, 32 inflammation-associated proteins were identified based on Venn diagram analysis, PPI network construction, and compound–drug–target inflammation network analysis. These proteins were further refined to 15 candidates belonging to the most significantly enriched functional cluster, namely nuclear receptor signaling. Protein structural availability was subsequently evaluated using the Protein Data Bank (PDB), and only targets with experimentally resolved structures containing co-crystallized ligands were retained, yielding five final docking targets. These five proteins were associated with 24 reference drug ligands. Fingerprint-based structural similarity screening between the reference ligands and the curated seaweed metabolite library led to the selection of six candidate metabolites (SW005, SW010, SW048, SW052, SW088, and SW107). These prioritized metabolites were then subjected to structure-based molecular docking against the five selected protein targets using the corresponding native co-crystallized ligands to define the binding sites.

#### 2.6.2 Protein preparation

Three-dimensional (3D) crystal structures of target proteins were retrieved from the RCSB Protein Data Bank (accessed December 2024) [49]. Only proteins with an available crystal structure and a co-crystallized ligand were retained, using the native ligand to define the reference binding-site coordinates. Non-essential components, including co-crystallized ligands, heteroatoms, and water molecules, were removed using BIOVIA Discovery Studio Client 2025. Polar hydrogens and Kollman charges were added, and files were converted to PDBQT format in AutoDock Tools version 1.5.7 [50]. For the GR structure (PDB 4P6X), the co-crystallized ligand is annotated as cortisol, because cortisol and hydrocortisone are identical chemical entities, hydrocortisone was used interchangeably as the reference ligand in subsequent docking and MD simulations.

#### 2.6.3 Ligand preparation

Seaweed-derived metabolites exhibiting high structural similarity to anti-inflammatory drugs were selected for docking. Their 2D structures were retrieved from PubChem [32] in SDF format and converted into 3D conformations using Open Babel [51] in PDB format. Energy minimization was performed using the Optimized Geometry tool in Avogadro version 1.2.0 [52], and further optimization was applied via the Clean Geometry module in BIOVIA Discovery studio client 2025. Reference drugs and co-crystallized ligands were included as positive controls and prepared using the same workflow. All ligands were prepared by adding polar hydrogens, Kollman charges, and Gasteiger charges. Rotatable bonds were defined, and the structures were saved in PDBQT format [53].

#### 2.6.4 Molecular docking

Docking was carried out using AutoDock Vina version 1.2.0 [54] under Linux. Targeted docking was performed because the target proteins contain a well-characterized binding pocket defined by the co-crystallized ligand in the template PDB structure [55]. Docking grids were therefore centered on the coordinates of the native ligand, using a grid box of 20 × 20 × 20 Å. Docking parameters included an exhaustiveness value of 64 and a maximum of 20 docking modes per ligands, and each docking experiment was performed in ten independent replicates. For each ligand–protein pair, the binding affinity used for comparative ranking was defined as the most frequently observed binding affinity (mode value) obtained from the ten docking replicates. Protocol validation was conducted by redocking the co-crystal ligand into its original binding site using the same grid setting and docking parameters. Predicted binding affinities (ΔG, kcal/mol) and corresponding docking poses were subsequently used for comparative analysis and prioritization. Docking conformations and intermolecular interactions were visualized in BIOVIA Discovery Studio Client 2025, with interaction analysis focusing on hydrogen bonding, hydrophobic interactions, alkyl interactions, and π-π interactions.

### 2.7 Molecular dynamics (MD) simulations

To investigate the conformational stability of protein–ligand complexes, all-atom MD simulations were conducted with the AMBER22 software package [56]. The simulated systems included GR proteins, each in complex with selected seaweed metabolites (SW052) or hydrocortisone, a reference compound. The protein parameters were described by the FF19SB force field [57], while the ligand parameters were generated using the GAFF2 force field [58]. The partial atomic charges of ligands were derived using the Restrained Electrostatic Potential (RESP) fitting procedure. Each complex was then solvated in a 12 Å padded truncated cubic box with the explicit TIP3P water model [59], and counterions were added to achieve charge neutrality.

The prepared systems were subjected to a standardized MD protocol. First, a two-step energy minimization was performed to remove steric clashes, consisting of 5000 steps of steepest descent followed by 5000 steps of conjugate gradient minimization. The systems were then gradually heated to 310 K under constant volume (NVT) conditions using the Langevin thermostat [60]. Subsequently, the systems were equilibrated in the isothermal-isobaric (NPT) ensemble at 310 K and 1 atm, utilizing the Berendsen barostat [61] for pressure control. For all simulations, the Particle Mesh Ewald method [62] was employed for the efficient calculation of long-range electrostatic interactions under periodic boundary conditions. The SHAKE algorithm [63] was used to constrain the lengths of all bonds involving hydrogen atoms, which allows for an integration time step of 2 fs. Following equilibration, production simulations were performed for each system under the NPT ensemble until reaching 500 ns. The MD trajectories were analyzed with the CPPTRAJ program [64]. To assess the overall stability and structural integrity of the complexes, the root-mean-square deviation (RMSD) of the protein backbone, the radius of gyration (R_g_) of the protein, and the number of intermolecular hydrogen bonds (# H-bonds) were computed over the course of the simulation. Additionally, the Molecular Mechanics/Poisson-Boltzmann Surface Area (MM/PBSA) and Molecular Mechanics/Generalized Born Surface Area (MM/GBSA) methods were applied using the MMPBSA.py script [65] in AmberTools to estimate the binding affinity. This calculation was performed on snapshots extracted from the production trajectory to obtain the total binding free energy and to decompose this energy on a per-residue basis, identifying key residues involved in ligand binding. All MD simulations for the GR–SW052 and GR-hydrocortisone complex were performed in three independent trajectories to assess reproducibility.

### 2.8 ADMET and skin sensitization analysis

Pharmacokinetic properties, drug-likeness, and toxicity of seaweed-derived metabolites were assessed using the SwissADME web server (accessed February 2025) [66], based on Lipinski’s Rule of Five [67]. Assessed parameters included physicochemical properties, lipophilicity, pharmacokinetics, water solubility, and drug-likeness. Toxicity was predicted using the ProTox 3.0 web server (https://tox.charite.de/protox3/; accessed March 2025) [68], which estimates organ-specific toxicity, toxicological endpoints, and metabolism. Skin permeability, and skin sensitization were predicted using the pkCSM (https://biosig.lab.uq.edu.au/pkcsm/; accessed March 2025) [69] to provide additional insight into systemic exposure and safety-related distribution properties. For comparative benchmarking, ADMET predictions for dexamethasone were generated using the same computational tools and parameters and are reported alongside the seaweed-derived metabolites in Table 4. These *in silico* assessments provided preliminary insights into the pharmacokinetic behavior and systemic and topical safety profiles of candidate metabolites.

### 2.9 In vitro biological activity

#### 2.9.1 Cell culture and treatment

Murine macrophages RAW264.7 (ATCC® TIB-71™) were cultured in Dulbecco’s Modified Eagle’s Medium (DMEM) supplemented with 10 % heat-inactivated fetal bovine serum (FBS) and maintained at 37 °C in a 5 % CO_2_ incubator. The cells were plated at a density of 1 × 10^5^ cells per well in 96-well plates and incubated under the same conditions for 24 h. Then, the cells were exposed to 1000 µg/mL lipopolysaccharide (LPS) from *Escherichia coli* O111:B4 (Sigma-Aldrich) and treated with or without seaweed lipophilic extract and active compounds for 24 h. The supernatant was then collected to measure nitric oxide level, while the cells were utilized to assess cell viability.

#### 2.9.2 Cell viability assay

Cell viability was estimated by 3-(4,5-dimethylthiazol-2-yl)-2,5-diphenyl tetrazolium bromide (MTT) assay. After treatment, the supernatant was removed, and the cells were stained with 100 µL of 0.1 mg/mL MTT at 37 °C in a 5 % CO_2_ for 2 h. After discarding the media, formed formazan crystals in the cells were dissolved with 100 µL of DMSO. The absorbance was measured using a microplate reader at 570 nm. The percentage of cell viability was calculated by the following equation. Non-toxic concentrations of the samples that exhibited over 70 % viability compared to the untreated group were selected to assess anti-inflammatory activity.

% viability = 100 × A _treated group_/A _LPS-induced group_

#### 2.9.3 Anti-inflammatory activity assay

Nitrite, a stable form of nitric oxide, was assessed by Griess reagent. The supernatant (100 µL) of the treated cells was transferred to a new well of a 96 well plate. Nitric oxide levels in supernatant were then mixed with an equal volume of Griess reagent (1 % (w/v) sulfanilamide and 0.1 % (w/v) naphthyl ethylenediamine dihydrochloride in 2.5 % (v/v) phosphoric acid) and incubated at room temperature for 10 min. The absorbance was measured using a microplate reader at 540 nm. The percentage of nitric oxide inhibition was calculated by the following equation, and the nitrite concentration was also calculated using a nitrite calibration curve.

% nitric oxide inhibition = 100 × (A _LPS-induced group_ – A _treated group_)/ A _LPS-induced group_

A _LPS-induced group_ was the cells induced with LPS only, and A _treated group_ was the cells treated with the samples with or without LPS.

### 2.10 Statistical analysis

All *in vitro* assays were performed in triplicate (n = 3), and the results were expressed as mean ± standard deviation (SD). Statistical differences among experimental groups were analyzed using one-way analysis of variance (ANOVA), followed by Tukey’s post hoc test, using IBM SPSS Statistics 23. A p-value < 0.05 was considered statistically significant.

## 3 Results

### 3.1 Cheminformatics screening of seaweed-derived metabolites

A total of 112 steroidal metabolites were curated from three marine macroalgae, including *S. polycystum*, *G. fisheri*, and *C. lentillifera*, as reported in a previous phytochemical investigation [30]. All compounds were structurally verified and annotated with canonical SMILES retrieved from major repositories (PubChem, ChemSpider, ChEMBL, SpectraBase, and the Seaweed Metabolite Database) to ensure chemical accuracy and cross-database consistency. For reference comparison, 7276 approved drugs with valid SMILES were extracted from DrugBank (v5.1.10) and used to generate a comprehensive structural dataset suitable for cheminformatics and molecular modeling. The combined chemical library was standardized for downstream similarity mapping, pharmacophore modeling, and receptor-based docking analyses. This validated dataset provided a robust computational foundation for identifying seaweed-derived steroidal scaffolds with potential macromolecular interactions toward the GR and other inflammation-related targets.

### 3.2 Fingerprint-based molecular similarity screening

**Fig. 1:**
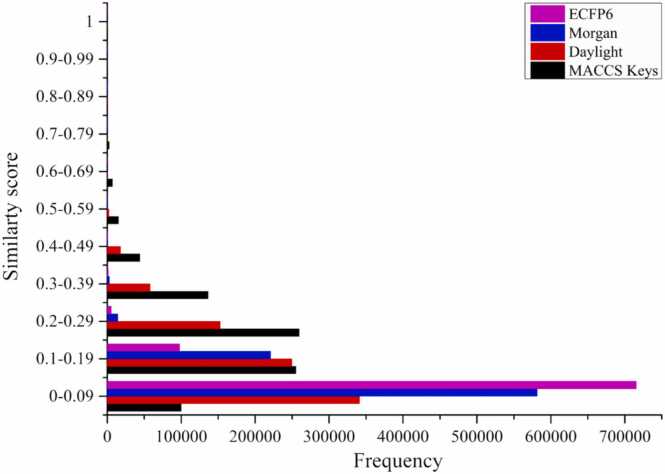
*In silico* distribution of Tanimoto similarity coefficients (Tc) between 112 seaweed-derived metabolites and 7276 DrugBank compounds using four fingerprinting methods, including MACCS Keys (black), Daylight (red), Morgan (blue), and ECFP6 (magenta). Most pairs showed low similarity (Tc < 0.3), while a small subset (Tc ≥ 0.85) indicated high predicted structural resemblance to approved drugs.

Pairwise *in silico* similarity analyses were performed between 112 seaweed-derived steroidal metabolites and 7276 approved drugs from DrugBank using four complementary fingerprinting algorithms, including MACCS keys, Morgan, Daylight, and ECFP6. A similarity threshold of Tc ≥ 0.85 was applied to identify high-confidence structural analogs. A total of 337 metabolite–drug pairs were obtained, among which 68 metabolites (60.7 %) exhibited high structural similarity to at least one approved drug (Table S1). Metabolites exhibiting similarity values between Tc = 0.70 and 0.84, which fell just below the applied threshold, are summarized in Table S2. Under the union-based fingerprint selection scheme, the MACCS keys method found the highest number of significant matches (68), followed by Morgan, Daylight, and ECFP6 fingerprints (Fig. 1). This distribution shows that key-based fingerprints were particularly effective in capturing conserved structural features characteristic of steroidal scaffolds, while circular and path-based fingerprints provided complementary but fewer matches. The high proportion of seaweed metabolites displaying strong similarity to approved drugs suggests their chemical feasibility and pharmacophore resemblance to existing therapeutic. These results support the prioritization of steroid-like seaweed metabolites for downstream therapeutic classification, target association analysis, and macromolecular docking with the GR.

### 3.3 Therapeutic classification of structurally similar drugs

**Fig. 2:**
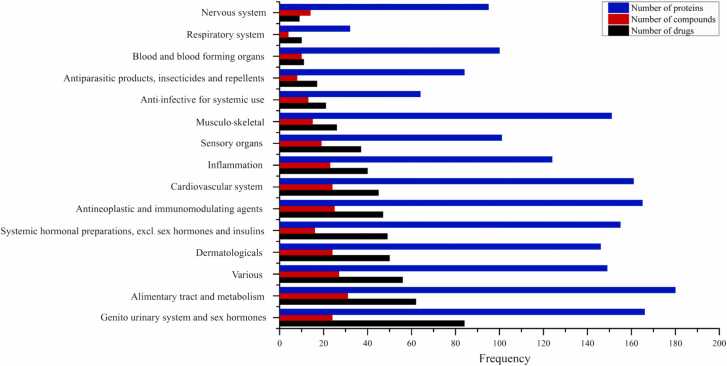
*In silico* classification of structurally similar drugs, seaweed-derived metabolites, and associated protein targets across WHO ATC classes, including the additional inflammation group. Bars represent the number of drugs (black), seaweed metabolites (red), and target proteins (blue).

Given that many seaweed-derived steroidal metabolites exhibited high structural similarity to approved drugs, their therapeutic classifications were analyzed to determine potential pharmacological associations. A total of 152 approved drugs identified through *in silico* similarity mapping (Tc ≥ 0.85) were categorized according to the WHO Anatomical Therapeutic Chemical (ATC) system, encompassing all 14 major pharmacological classes and an additional functional “inflammation” category. The predominant categories were the genito-urinary system and sex hormones (84 drugs, 55.3 %), the alimentary tract and metabolism (62 drugs, 40.8 %), and dermatological preparations (50 drugs, 32.9 %) (Fig. 2, Table 1). Forty drugs were functionally annotated as anti-inflammatory agents, including both steroidal (e.g., hydrocortisone, medrysone) and non-steroidal anti-inflammatory drugs (NSAIDs). However, most of the high-confidence structural analogs aligned with steroidal frameworks, suggesting that seaweed-derived steroidal metabolites share greater pharmacophoric similarity with glucocorticoid-type scaffolds than with NSAID chemotypes. This pharmacological clustering provides a rational basis for prioritizing steroid-like seaweed metabolites, particularly those structurally analogous to corticosteroids, for subsequent target prediction, macromolecular docking, and GR binding evaluation.

**Table 1:** Distribution of structurally similar drugs identified through Tanimoto similarity (Tc ≥0.85), their corresponding seaweed-derived metabolites, and associated protein targets classified by Anatomical Therapeutic Chemical (ATC) and inflammation categories.

**Classification**	**No. of structurally similar drugs**	**No. of seaweed-derived metabolites**	**No. of protein targets**	**Inflammation-related protein targets**	**% of inflammation-related proteins**
Genito urinary system and sex hormones	84	24	166	80	48.19
Alimentary tract and metabolism	62	31	180	96	53.33
Various	56	27	149	84	56.38
Dermatologicals	50	24	146	82	56.16
Systemic hormonal preparations, excl. sex hormones and insulins	49	16	155	93	60.00
Antineoplastic and immunomodulating agents	47	25	165	111	67.27
Cardiovascular system	45	24	161	83	51.55
Inflammation	40	23	124	124	100.00
Sensory organs	37	19	101	65	64.36
Musculo-skeletal system	26	15	151	87	57.62
Anti-infective for systemic use	21	13	64	60	93.75
Antiparasitic products, insecticides and repellents	17	8	84	35	41.67
Blood and blood forming organs	11	10	100	58	58.00
Respiratory system	10	4	32	32	100.00
Nervous system	9	14	95	74	77.89

**Note.** Inflammation-related protein targets = overlap with the inflammation set; **%** = overlap ÷ targets × 100.

### 3.4 Identification of inflammation-related protein targets

**Fig. 3:**
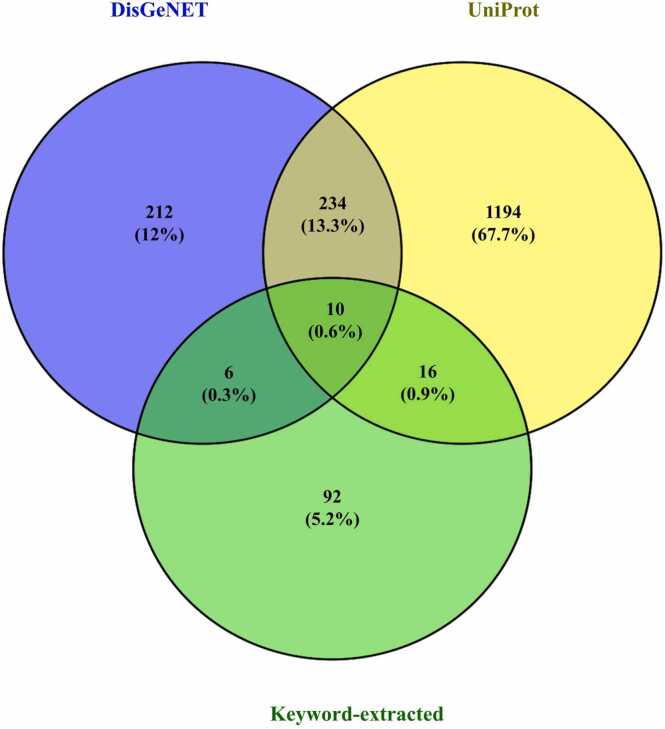
*In silico* Venn diagram showing the overlap of inflammation-associated proteins retrieved from DisGeNET, UniProt, and keyword-extracted datasets.

Following the therapeutic classification, inflammation-associated protein targets were identified to establish potential molecular links with the predicted drug-like metabolites. *In silico* data extraction yielded an initial pool of 124 proteins retrieved using inflammation-related keywords from the DisGeNET and UniProt databases. Among these, 16 proteins were identified in DisGeNET and 26 proteins in UniProt, with 10 overlapping entries (Fig. 3, Table S3). After eliminating redundant records, 32 unique inflammation-associated proteins were retained as high-confidence targets for subsequent network and docking analyses. These proteins included key regulators such as prostaglandin-endoperoxide synthase 2 (COX-2; PTGS2), glucocorticoid receptor (GR; NR3C1), peroxisome proliferator-activated receptor α (PPARA), estrogen receptor 1 (ESR1), and 11β-hydroxysteroid dehydrogenase 1 (HSD11B1). The overlap between DisGeNET and UniProt supported the cross-database consistency and biological relevance of these predicted targets, which were subsequently used to construct protein–protein and compound–target interaction networks to elucidate their macromolecular connectivity in inflammatory signaling.

### 3.5 Network-based analyses

#### 3.5.1 Protein–protein interaction (PPI) network

**Fig. 4:**
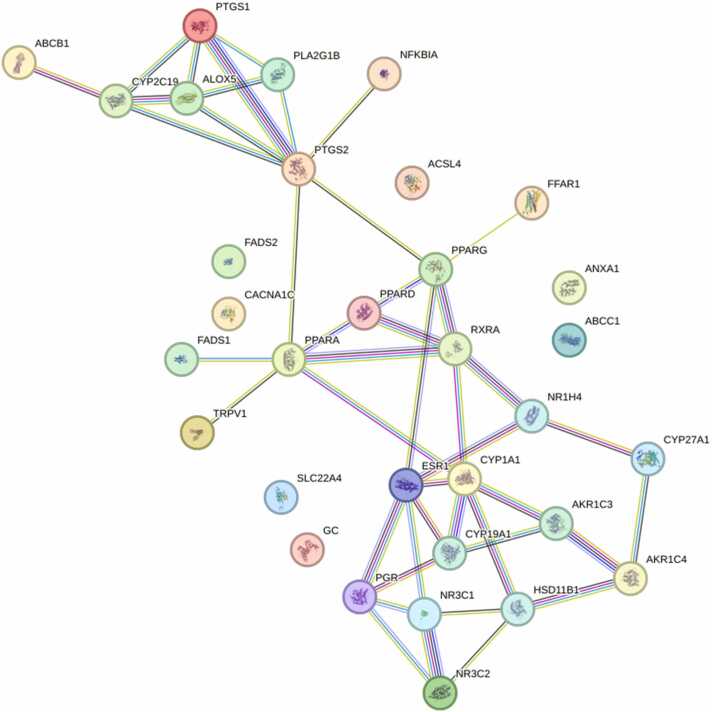
*In silico* protein–protein interaction (PPI) network of 32 predicted inflammation-related targets.

To explore the molecular interconnections among inflammation-related targets, a PPI network was generated using STRING v12.0 (confidence score ≥ 0.7). The resulting network consisted of 25 nodes and 88 edges, representing high-confidence interactions among the 32 predicted proteins. Topological analysis identified prostaglandin-endoperoxide synthase 2 (PTGS2) as the principal hub (degree = 7), consistent with its central role in prostaglandin biosynthesis and inflammatory signaling. Other highly connected nodes, including PPARA, CYP1A1, and ESR1, suggest potential cross-regulatory links between metabolic and hormonal pathways (Fig. 4, Table 2). Collectively, these findings indicate that inflammation is coordinated by multiple interconnected regulatory proteins, with PTGS2 as a dominant node, while other receptors may serve as crosstalk points relevant to the activity of seaweed-derived steroidal metabolites.

**Table 2:** Topological properties of inflammation-associated proteins in the protein–protein interaction (PPI) network. Metrics include degree, betweenness centrality, and closeness centrality.

**No.**	**UniProt**	**Gene**	**Degree**	**Betweenness Centrality**	**Closeness Centrality**
**1**	P35354	PTGS2	7	0.42	0.45
**2**	Q07869	PPARA	6	0.34	0.49
**3**	P04798	CYP1A1	6	0.25	0.47
**4**	P03372	ESR1	6	0.21	0.46
**5**	P37231	PPARG	5	0.27	0.46
**6**	P19793	RXRA	5	0.10	0.44
**7**	P33261	CYP2C19	4	0.08	0.33
**8**	P28845	HSD11B1	4	0.08	0.37
**9**	P04150	NR3C1	4	0.03	0.35
**10**	P06401	PGR	4	0.02	0.35
**11**	P11511	CYP19A1	4	0.02	0.38
**12**	P09917	ALOX5	4	0.00	0.33
**13**	P23219	PTGS1	4	0.00	0.33
**14**	Q96RI1	NR1H4	3	0.07	0.37
**15**	P42330	AKR1C3	3	0.03	0.35
**16**	P17516	AKR1C4	3	0.02	0.30
**17**	Q03181	PPARD	3	0.01	0.39
**18**	P08235	NR3C2	3	0.00	0.30
**19**	P04054	PLA2G1B	3	0.00	0.32
**20**	Q02318	CYP27A1	2	0.01	0.30
**21**	O60427	FADS1	1	0	0.33
**22**	Q8NER1	TRPV1	1	0	0.33
**23**	O14842	FFAR1	1	0	0.32
**24**	P25963	NFKBIA	1	0	0.32
**25**	P08183	ABCB1	1	0	0.25

#### 3.5.2 Compound–drug–target inflammation network

**Fig. 5:**
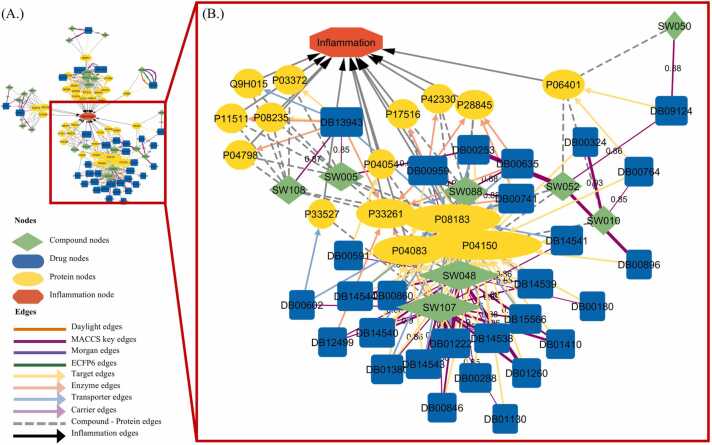
*In silico* compound–drug–target–inflammation network generated in Cytoscape v3.10.2. (A) Global view of the integrated network. (B) Enlarged subnetwork highlighting nuclear receptor-related interactions. Node size reflects degree centrality, and colors denote compound, drug, or target categories.

To integrate the chemical and biological data, a compound–drug–target–inflammation network was constructed in Cytoscape v3.10.2, encompassing 90 nodes and 316 edges (Fig. 5A). Four major clusters were observed. The largest was enriched in nuclear receptor signaling, including steroid hormone receptors and peroxisome proliferator-activated receptors (PPARs) (Fig. 5B). Within this cluster, 8 seaweed metabolites (SW005, SW010, SW048, SW050, SW052, SW088, SW107, and SW108) displayed high degree centrality and multiple predicted connections to nuclear-receptor nodes. These results suggest that several seaweed-derived steroidal metabolites may act as putative interactors of nuclear receptors, particularly glucocorticoid and PPAR-related pathways, thereby contributing to anti-inflammatory modulation. This systems-level network analysis provides a mechanistic rationale for prioritizing the identified steroidal metabolites, especially 11α-hydroxyprogesterone (SW052), for subsequent macromolecular docking and MD simulations.

### 3.6 Target prioritization for molecular docking

To identify suitable macromolecular targets for receptor–ligand interaction studies, *in silico* prioritization was conducted using the 32 inflammation-associated proteins identified earlier. Among these, 15 proteins were shortlisted based on their nuclear receptor-mediated functions, clinical druggability, and metabolite–drug similarity inferred from the compound–drug–target–inflammation network. Further refinement using the specificity of targeted glucocorticoid drug, the availability of experimentally determined PDB structures, and the presence of co-crystallized ligands yielded five final protein targets for docking evaluation. On the ligand side, 24 structurally similar drugs and six seaweed-derived steroidal metabolites were retained (Table S4). Most of the prioritized targets were linked to steroidal anti-inflammatory drugs rather than NSAIDs, reinforcing the hypothesis that seaweed-derived steroidal metabolites preferentially engage steroid hormone-related pathways, including the glucocorticoid receptor axis. These similarity- and network-based selections suggest that marine-derived steroidal metabolites may interact with targets associated with glucocorticoid signaling, thereby supporting their potential significance in inflammatory regulation.

### 3.7 Molecular docking and interaction profiling

Using the integrated prioritization strategy, six seaweed-derived steroidal metabolites (SW005, SW010, SW048, SW052, SW088, and SW107) were docked against five inflammation-related protein targets and directly compared with reference anti-inflammatory drugs using identical co-crystallized binding sites. The resulting docking profiles (Table 3) enabled a comparative evaluation of ligand accommodation and predicted binding affinities between seaweed-derived metabolites and clinically relevant reference compounds, providing a foundation for subsequent target-focused analysis. Because the glucocorticoid receptor (GR; PDB ID: 4P6X) plays a central role in inflammatory regulation and was consistently enriched across network-based analyses, subsequent docking investigations were focused on the GR. Clinically used glucocorticoids were included as reference ligands to provide pharmacological context and benchmarking. Hydrocortisone and medrysone were selected as primary reference ligands due to their well-established GR agonist activity, while dexamethasone was additionally incorporated as a potent synthetic glucocorticoid to enable comparison across clinically relevant GR-binding profiles under an identical docking protocol. Docking calculations were performed using the native co-crystallized ligand to define the canonical GR ligand-binding pocket, ensuring consistent evaluation of ligand accommodation and interaction patterns.

**Table 3:** Mode binding affinities (ΔG, kcal/mol) from ten docking replicates and key interacting residues and hydrogen bond lengths (Å) of prioritized seaweed-derived metabolites and reference anti-inflammatory ligands docked against inflammation-related targets.

**Protein**	**Grid center coordinates (Å)**	**Ligands**	**Chemical structure**	**Binding affinity (kcal/mol)**	**Interactions**
Glucocorticoid receptor (GR; PDB: 4P6X)	4.231 × 32.554 × -6.139	Control ligands			
		Medrysone (Fingerprint ligand)		-11.6	**Hydrogen Bonds**: GLN570 (2.32), ARG611 (2.24) **Hydrophobic Interactions**: CYS736 (3.67) **Alkyl interactions**: MET604 (4.70), ALA605 (3.54), LEU608 (4.14), MET646 (4.20, 5.36), LEU732 (5.23, 5.45)
		Hydrocortisone (Co-crystallized ligand)		-11.4	**Hydrogen Bonds**: GLN570 (2.31), ARG611 (2.02) **Hydrophobic Interactions**: CYS736 (3.68, 5.22) **Alkyl interactions**: MET601 (5.42), MET604 (4.65), MET646 (5.28), LEU732 (5.13)
		Dexamethasone		-11.1	**Hydrogen Bonds**: ASN564 (2.04), ARG611 (2.32) **Alkyl interactions**: TRP600 (5.22), MET601 (5.38, 5.44), MET604 (4.40), MET646 (5.32), LEU732 (4.66, 5.09)
		Seaweed-derived metabolite			
		SW052		-11.6	**Hydrogen Bonds**: ASN564 (2.87), GLN570 (2.34), ARG611 (2.07) **Hydrophobic Interactions**: CYS736 (3.64, 5.24) **Alkyl interactions**: TRP600 (5.40), MET601 (5.20, 5.33, 5.41), MET604 (4.72), MET646 (5.05), LEU732 (5.14)
Progesterone receptor (PGR; PDB: 1A28)	21.633 × 11.354 × 60.450	Control ligands			
		Mometasone (Fingerprint ligand)		-11	**Hydrogen Bonds:** ARG766 (2.08) **Hydrophobic Interactions:** CYS891 (3.14) **Alkyl interactions:** LEU715 (4.86), TRP755 (5.21), MET756 (4.71, 5.45), MET759 (3.87), PHE794 (4.46), LEU797 (4.75), TYR890 (4.04)
		Progesterone (Co-crystallized ligand)		-11.5	**Hydrogen Bonds:** GLN725 (2.62), ARG (2.27) **Hydrophobic Interactions:** CYS891 (3.40,3.68,4.89) **Alkyl interactions:** LEU718 (4.93), MET756 (5.38), MET759 (4.16)
		Seaweed-derived metabolite			
		SW010		-11	**Alkyl interactions:** LEU715 (5.13), LEU718 (5.24), LEU721 (5.42), PHE778 (5.07), PHE794 (4.78), LEU797 (4.94), LEU887 (5.50), TYR890 (4.57), CYS891 (3.79, 4.87)
Corticosteroid 11-beta-dehydrogenase isozyme 1 (HSD11B1; PDB: 2ILT)	63.537 × 103.522 × 45.956	Control ligands			
		Methylprednisolone (Fingerprint ligand)		-9.1	**Hydrogen Bonds**: SER169 (2.84) **Hydrophobic Interactions**: GLY41 (3.68) **Alkyl interactions**: LYS44 (4.78), ILE121 (4.77), ALA223 (4.38)
		2-(2-chloro-4-fluorophenoxy)-2-methyl-n-[1r,2 s,3 s,5 s,7 s)-5-(methylsulfonyl)-2-adamantyl] propenamide (Co-crystallized ligand)		-9.8	**Hydrogen Bonds**: ILE46 (2.15), ASN119 (2.11), TYR183 (2.99, 3.91) **Alkyl interactions**: ALA172 (4.53) **Pi-pi T-shaped interactions:** TYR177 (5.61)
		Seaweed-derived metabolite			
		SW048		-9.9	**Hydrophobic Interactions**: LEU217 (3.56, 4.49) **Alkyl interactions**: ILE46 (4.31, 5.37), LEU171 (4.36), TYR177 (3.75, 4.13), ILE218 (5.05), ALA223 (3.69) **Unfavorable acceptor-acceptor:** SER170 (2.73)
Cytochrome P450 2C19 (CYP2C19; PDB: 4GQS)	-80.867 × 20.981 × -43.62	Control ligands			
		Budesonide (Fingerprint ligand)		-8.4	**Hydrogen Bonds:** ASN204 (2.26), ASP293 (1.94) **Alkyl interactions:** LEU201 (4.63), VAL113 (4.40), PHE114 (4.98), LEU366 (4.71)
		(4-hydroxy-3,5-dimethylphenyl)(2-methyl-1-benzofuran-3-yl)methanone(Co-crystallized ligand)		-8.5	**Alkyl interactions:** LEU102 (4.47), ALA103 (5.00), VAL113 (3.80, 5.10), ILE205 (4.70), LEU233 (5.39), ALA297 (3.86, 5.32), LEU366 (4.08), PHE476 (5.09) **Pi-Sigma:** VAL208 (2.44, 4.59, 4.79) **Amide-Pi Stacked:** ASN204 (5.39), GLY296 (4.81)
		Seaweed-derived metabolite			
		SW107		-9.4	**Hydrogen Bonds:** ARG97 (2.89), ASN204 (2.83), ASP293 (2.11, 2.56) **Hydrophobic Interactions:** ILE205 (2.77), ALA297 (2.69) **Alkyl interactions:** ILE362 (3.63), LEU366 (3.99), PHE476 (5.46)
Aldo-keto reductase family 1 member C3 (AKR1C3; PDB: 1S2A)	24.770 × -27.254 × 60.059	Control ligands			
		Methylprednisolone (Fingerprint ligand)		-10.2	**Hydrogen Bonds**: LYS270 (2.56, 4.55) **Alkyl interactions**: TYR24 (5.05), TYR216 (4.78, 5.23, 5.25), TRP227 (5.11), LEU268 (4.82)
		Indomethacin (Co-crystallized ligand)		-9.8	**Hydrogen Bonds**: GLN222 (2.27) **Hydrophobic Interactions**: SER217 (2.39) **Alkyl interactions**: TYR24 (3.47), TYR55 (4.87), MET120 (4.91), PHE311 (3.87) **Pi-pi T-shaped interactions:** TRP86 (5.21, 5.49), HIS117 (4.92), TRP227 (5.01), PHE306 (5.15, 5.37)
		Seaweed-derived metabolite			
		SW048		-10.6	**Hydrogen Bonds**: TYR216 (2.31, 3.00), LYS270 (2.59) **Hydrophobic Interactions**: TYR55 (2.99, 4.42, 4.48) **Alkyl interactions**: LEU54 (5.18), TRP86 (4.91), HIS117 (4.64), TRP227 (4.75), PHE311 (4.99)

Note: The complete set of all ten replicate docking scores for each ligand–protein combination has now been provided in Table S5-S9.

Among the prioritized seaweed-derived steroidal metabolites, SW052 (11α-hydroxyprogesterone) consistently demonstrated the most favorable predicted binding profile toward the GR and was therefore selected for detailed docking and interaction profiling. SW052 yielded a predicted binding affinity of −11.6 kcal/mol, identical to that of medrysone (−11.6 kcal/mol) and closely comparable to hydrocortisone (−11.4 kcal/mol), whereas dexamethasone exhibited a slightly less favorable docking score (−11.1 kcal/mol) under the same conditions (Table 3). The ≤ 0.2 kcal/mol difference observed between SW052 and the reference ligands lies within the expected uncertainty of empirical docking scoring functions, indicating comparable predicted binding strong rather than functional equivalence. In addition to docking scores, hydrogen-bond distances were evaluated to further characterize interaction quality. Hydrogen bonds with donor–acceptor distances below approximately 3.5 Å are generally regarded as geometrically favorable and indicative of stable ligand–receptor interactions, as commonly applied in structure-based docking analyses. As summarized in Table 3, the hydrogen-bond distances observed for SW052 and the reference ligands fall within this favorable range, supporting comparable binding geometries across the evaluated complexes.

**Fig. 6:**
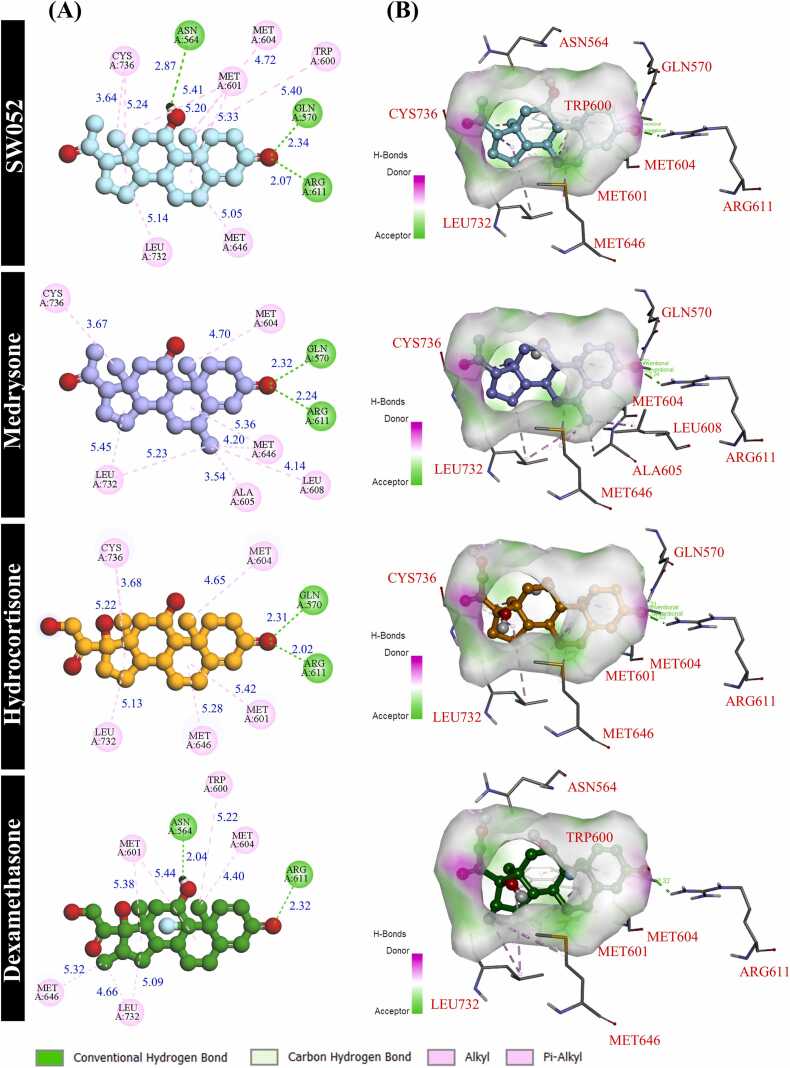
(A) 2D interaction diagrams for GR complexes with SW052, medrysone, hydrocortisone and dexamethasone showing H-bond, hydrophobic and alky interactions; (B) 3D binding-surface views highlighting donor/acceptor regions in the GR ligand binding domain.

Interaction profiling revealed that SW052 occupied the canonical GR ligand-binding pocket and shared a conserved interaction pattern with reference glucocorticoids. Key hydrogen bonds were observed with GLN570 and ARG611, residues known to be critical for GR ligand recognition. In addition, SW052 formed hydrogen bonds with ASN564 (2.87 Å), GLN570 (2.34 Å), and ARG611 (2.07 Å), together with hydrophobic anchoring at CYS736 (3.64 and 5.24 Å). Alkyl interactions involving TRP600, MET601, MET604, MET646, and LEU732 further contributed to stabilization of the ligand-binding pocket (Table 3, Fig. 6). In comparison, medrysone formed hydrogen bonds with GLN570 (2.32 Å) and ARG611 (2.24 Å), accompanied by hydrophobic and alkyl interactions involving CYS736, MET604, ALA605, LEU608, MET646, and LEU732. Hydrocortisone displayed a similar interaction pattern, forming hydrogen bonds with GLN570 (2.31 Å) and ARG611 (2.02 Å), along with hydrophobic interactions at CYS736 and alkyl contacts with MET601, MET604, MET646, and LEU732. The high-affinity synthetic glucocorticoid dexamethasone formed hydrogen bonds with ASN564 (2.04 Å) and ARG611 (2.32 Å), together with alkyl interactions involving TRP600, MET601, MET604, MET646, and LEU732, consistent with its established GR-binding mode.

Overall, these docking results support the prioritization of SW052 as a seaweed-derived steroidal metabolite with a favorable predicted GR-binding profile that closely resembles clinically used glucocorticoids at the level of binding site accommodation and residue-level interactions. However, as molecular docking provides a static and score-based approximation of ligand–receptor compatibility, further investigation of binding stability and conformational dynamics was required. Therefore, molecular dynamics simulations were subsequently performed to evaluate the temporal stability and dynamic behavior of the GR–SW052 complex.

### 3.8 In silico ADMET and skin sensitization prediction

To complement the molecular docking results, *in silico* ADMET and skin-sensitization analyses were performed to evaluate the predicted pharmacokinetic and safety profiles of SW052. The SwissADME predicted compliance with Lipinski’s Rule of Five and Veber’s criteria, suggesting favorable oral drug-likeness (Table 4). SW052 showed high gastrointestinal absorption, moderate lipophilicity (Consensus Log P = 3.09), and good aqueous solubility (Log S = –3.31). According to the SwissADME BBB model, SW052 was predicted to be BBB-permeant, whereas hydrocortisone and dexamethasone were classified as non-permeant (Table 4), indicating distinct predicted CNS-distribution tendencies rather than confirmed central activity. Toxicity predictions from ProTox-3.0 and pkCSM suggested a low-risk profile, with SW052 classified as non-hepatotoxic, non-neurotoxic, and non-mutagenic, and no predicted skin sensitization liability. In contrast, dexamethasone showed predicted hepatotoxic and neurotoxic signals in these models, while hydrocortisone remained largely inactive across the same endpoints. Metabolic liability analysis identified a potential interaction with CYP2C9 (active = 0.75) for SW052, whereas other CYP isoenzymes were predicted to be inactive. Dexamethasone, by comparison, was predicted to interact with additional CYP isoenzyme, suggesting a more complex predicted metabolic profile. The predicted skin permeability of SW052 (log Kp = –3.33) was higher than hydrocortisone and comparable to dexamethasone, supporting potential dermal applicability. Collectively, these *in silico* results suggest that SW052 possesses a drug-like and safety profile within the predictive range of approved glucocorticoids, while exhibiting distinct pharmacokinetic features that may be relevant for further preclinical evaluation in therapeutic or cosmaceutical contexts.

**Table 4:** Integrated SwissADME-, ProTox 3.0-, and pkCSM-predicted drug-likeness, pharmacokinetic, toxicity, and skin permeability/sensitization profile of the prioritized seaweed-derived metabolite 11α-hydroxyprogesterone (SW052), hydrocortisone (DB00741) and dexamethasone.

**Predicted drug-likeness and pharmacokinetic properties (SwissADME)**				
**Parameters**		**SW052**	**Hydrocortisone**	**Dexamethasone**
Physicochemical properties	Formula	C_21_H_30_O_3_	C_21_H_30_O_5_	C_22_H_29_FO_5_
	Molecular weight	330.46 g/mol	362.46 g/mol	392.46 g/mol
	No. H-bond acceptor	3	5	6
	No. H-bond donors	1	3	3
Lipophilicity	Log Po/w (XLOGP3)	2.36	1.61	1.94
	Log Po/w (WLOGP)	3.69	1.78	2.32
	Log Po/w (MLOGP)	3.07	1.39	1.62
	Log Po/w (SILICOS-IT)	3.59	2.47	2.58
	Consensus Log Po/w	3.09	1.89	2.15
Pharmacokinetics	GI absorption	High	High	High
	BBB permeant	Yes	No	No
Water Solubility	Log S (ESOL)	-3.31	-2.97	-2.80
	Solubility	1.62 × 10^−1^ mg/mL; 4.90 × 10^−4^ mol/l	2.22 × 10^−1^ mg/mL; 6.12 × 10^−4^ mol/l	6.25 × 10^−1^ mg/mL; 1.59 × 10^−3^ mol/l
	Class	Soluble	Soluble	Soluble
Drug likeness	Lipinski	Yes; 0 violation	Yes; 0 violation	Yes; 0 violation
	Veber	Yes	Yes	Yes
**Predicted toxicity, metabolism, and skin permeability/sensitization (ProTox 3.0, pkCSM)**				
**Classification**	**Target**	**Prediction and probability**		
Organ toxicity	Hepatotoxicity	Inactive (0.87)	Inactive (0.99)	Active (0.69)
	Neurotoxicity	Inactive (0.71)	Inactive (0.98)	Active (0.87)
Toxicity endpoints	Mutagenicity	Inactive (0.93)	Inactive (0.53)	Inactive (0.97)
	Nutritional Toxicity	Inactive (0.51)	Active (0.68)	Inactive (0.74)
Metabolism	CYP1A2	Inactive (1)	Inactive (1)	Inactive (0.76)
	CYP2C19	Inactive (0.57)	Inactive (1)	Inactive (0.87)
	CYP2C9	Active (0.75)	Inactive (0.93)	Active (0.56)
	CYP2D6	Inactive (0.98)	Inactive (0.87)	Inactive (0.63)
	CYP3A4	Inactive (0.62)	Inactive (1)	Active (0.71)
	CYP2E1	Inactive (1)	Inactive (0.99)	Inactive (0.98)
Skin Permeability (log Kp)		-3.33 cm/s	-7.37 cm/s	-3.972 cm/s
Skin Sensitization		No	No	No

### 3.9 Molecular dynamics simulations

#### 3.9.1 System stability

**Fig. 7:**
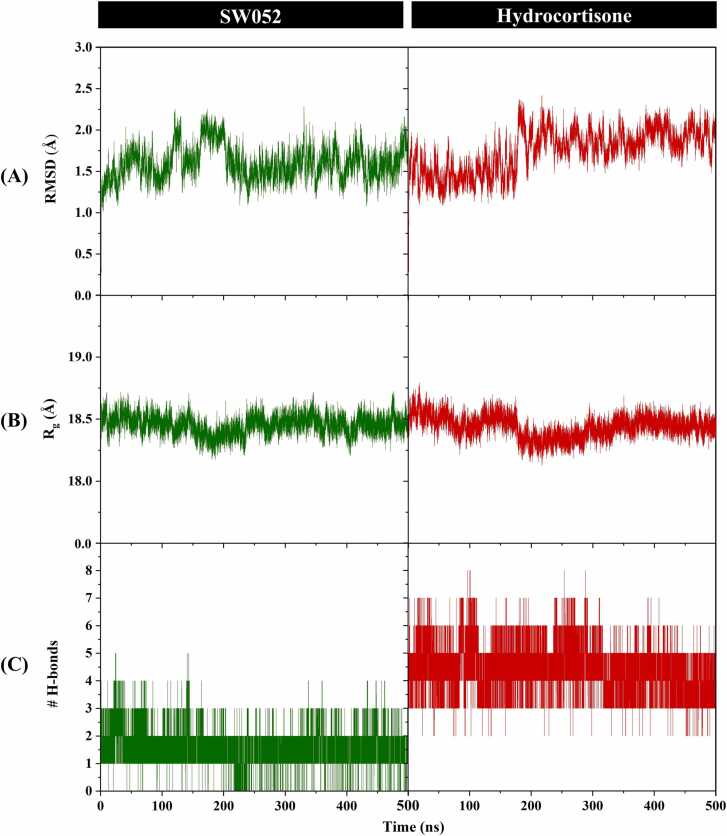
Time evolution of (**A**) RMSD, (**B**) radius of gyration (Rg), and (**C**) hydrogen bonding profiles for GR complexes with SW052 and hydrocortisone during 500 ns MD simulations.

The dynamic stability of GR complexes with SW052 and the reference ligand hydrocortisone was evaluated over 500 ns MD simulations. To ensure reproducibility, all simulations were performed in triplicate, and the resulting data demonstrated high consistency across independent trajectories (Fig. S1). Backbone RMSD profiles indicated that both systems reached equilibrium after approximately 200 ns (Fig. 7A). The GR–SW052 complex showed a stable trajectory with an average RMSD of ∼1.60 Å, which was slightly lower than ∼1.89 Å observed for the GR–hydrocortisone complex. This stability was further supported by comparable radius of gyration (R_g_) values ∼ 18.4 Å for both systems throughout the simulation (Fig. 7B), indicating no major compaction or unfolding. Hydrogen bond analysis revealed that hydrocortisone consistently maintained 2–8H-bonds with GR (averaging approximately 4) during the simulation, whereas SW052 formed 0–4H-bond (averaging approximately 1), suggesting a more persistent polar interaction network for hydrocortisone (Fig. 7C). Because the number of hydrogen bonds formed by hydrocortisone did not fall below two, the lower region of the H-bond plot appears unoccupied, reflecting sustained interaction rather than missing data. Overall, these results suggest that both ligands form dynamically stable complexes with GR. This was statistically confirmed by cluster analysis of the final 50 ns of the triplicate trajectories, where representative structures from the most populated clusters showed high conformational similarity. The population percentages for the top clusters were 22.0 %, 23.4 %, and 27.4 % for GR–SW052, and 30.1 %, 17.0 %, and 22.9 % for GR–hydrocortisone, indicating a highly consistent and reproducible binding orientation (Fig. S2).

#### 3.9.2 Binding free energy evaluation by MM/PBSA and MM/GBSA

To quantify the binding affinity, end-point free energy calculations were performed using the MM/PBSA and MM/GBSA methods based on 500 snapshots from the final 50 ns of the MD simulations. The GR-SW052 complex was predicted binding free energy a Δ*G*_bind_ of –34.36 ± 3.38 kcal/mol (MM/PBSA) and –45.40 ± 2.69 kcal/mol (MM/GBSA), whereas hydrocortisone show Δ*G*_bind_ of –46.93 ± 3.22 kcal/mol (MM/PBSA) and –55.06 ± 2.71 kcal/mol (MM/GBSA), respectively (Table 5). Energy decomposition analysis revealed that SW052 binding was primarily driven by strong van der Waals interactions (Δ*E*_vdW_ = –49.82 kcal/mol) and shape complementarity, partly offset by weaker electrostatic (Δ*E*_ele_ = –13.33 kcal/mol) and polar solvation components. Hydrocortisone, in contrast, showed both stronger Δ*E*_vdW_ (–53.45 kcal/mol) and Δ*E*_ele_ (–30.89 kcal/mol), consistent with its more extensive hydrogen bonding network (Fig. 7C). Taken together, these results suggest that hydrocortisone achieves more favorable predicted binding energetics through a combination of H-bond and electrostatic interaction, whereas SW052 interacts with GR primarily through hydrophobic and van der Waals–dominated interactions. These computational estimates should be interpreted as indicators of relative binding tendencies rather than quantitative measures of functional activity.

**Table 5:** Binding free energy components (kcal/mol) from MM/PBSA and MM/GBSA for GR–ligand complexes (SW052 and hydrocortisone).

**Energy Component(kcal/mol)**	**SW052 complex**	**Hydrocortisone complex**
**Gas term**		
Δ*E*_vdW_	−49.82 ± 2.27	−53.45 ± 2.86
Δ*E*_ele_	−13.33 ± 3.89	−30.89 ± 3.71
ΔE_MM_(=ΔE_vdW_ + ΔE_ele_)	−63.15 ± 4.19	−84.34 ± 3.26
**Solvation term** **PBSA**		
${\text{ΔG}}_{\text{sol}(\text{PBSA})}^{\text{ele}}$	32.84 ± 2.73	41.46 ± 2.09
${\text{ΔG}}_{\text{sol}(\text{PBSA})}^{\text{nonpolar}}$	−4.05 ± 0.08	−4.06 ± 0.06
Δ*G*_sol(PBSA)_	28.79 ± 2.74	37.41 ± 2.08
**GBSA**		
${\text{ΔG}}_{\text{sol}(\text{GBSA})}^{\text{ele}}$	23.74 ± 2.52	35.98 ± 1.84
${\text{ΔG}}_{\text{sol}(\text{GBSA})}^{\text{nonpolar}}$	−5.99 ± 0.15	−6.71 ± 0.12
Δ*G*_sol(GBSA)_	17.75 ± 2.51	29.28 ± 1.84
**Binding free energy**		
Δ*G*_bind(MM/PBSA)_	−34.36 ± 3.38	−46.93 ± 3.22
Δ*G*_bind(MM/GBSA)_	−45.40 ± 2.69	−55.06 ± 2.71

#### 3.9.3 Key binding residues

**Fig. 8:**
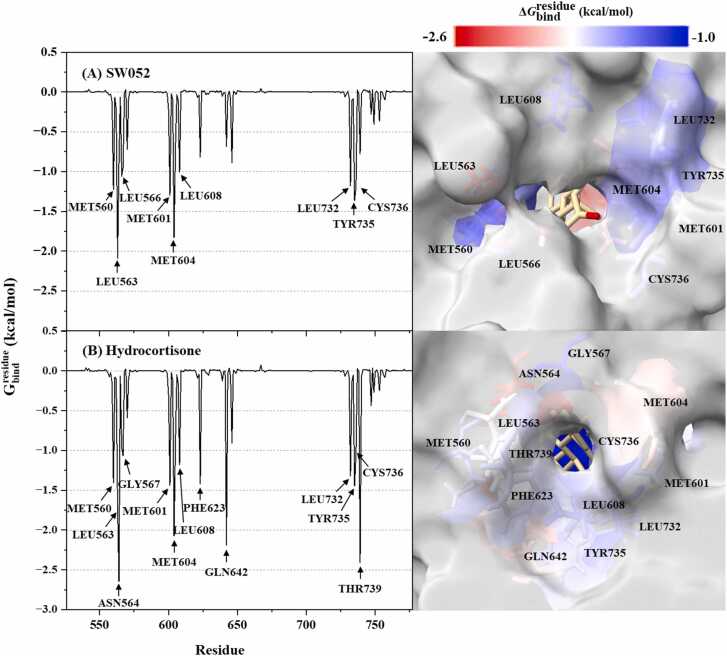
Per-residue binding free energy (kcal/mol) from MM/PBSA for GR in complex with (A) SW052 and (B) hydrocortisone. Only residues that stabilize binding by ≤ −1.0 kcal/mol are colored according to their ${\text{Δ}G}_{\text{bind}}^{\text{residue}}$ values in the active-site views at right. The residues with energy contribution ranging from −2.6 to −1.0 kcal/mol are shaded from red to blue, respectively. Binding poses are taken from the last snapshot of each 500 ns MD trajectory.

Per-residue free energy decomposition was performed using the MM/PBSA approach to identify the amino acid residues contributing most to the stabilization of GR–ligand complexes (Fig. 8). Residues with negative ${\text{Δ}G}_{\text{bind}}^{\text{residue}}$ values were considered to provide favorable contributions, while positive values indicated destabilizing effects. For the GR–SW052 complex, the major stabilizing residues included MET560, LEU563, LEU566, MET601, MET604, LEU608, LEU732, TYR735, and CYS736. The contributions were predominantly derived from nonpolar residues, indicating that SW052 binding is primarily stabilized by hydrophobic and van der Waals interactions within the ligand-binding domain. In contrast, the GR–hydrocortisone complex (DB00741) involved a broader and more diverse set of stabilizing hotspots, including MET560, LEU563, ASN564, GLY567, MET601, MET604, LEU608, PHE623, GLN642, LEU732, TYR735, CYS736, and THR739. Beyond the hydrophobic contacts shared with SW052, hydrocortisone formed additional hydrogen-bond and electrostatic interactions through ASN564, GLN642, and THR739, resulting in enhanced stabilization of the complex. This more extensive interaction network, encompassing both nonpolar and polar contributions, is consistent with the more negative binding free energy and stronger electrostatic component observed for hydrocortisone in the MM/PBSA and MM/GBSA analyses. Overall, these results unequivocally demonstrate that hydrocortisone exhibits a superior interaction profile within the GR-ligand-binding pocket, reflecting its established pharmacological potency.

### 3.10 In vitro biological activity

#### 3.10.1 Cell viability assay

**Fig. 9:**
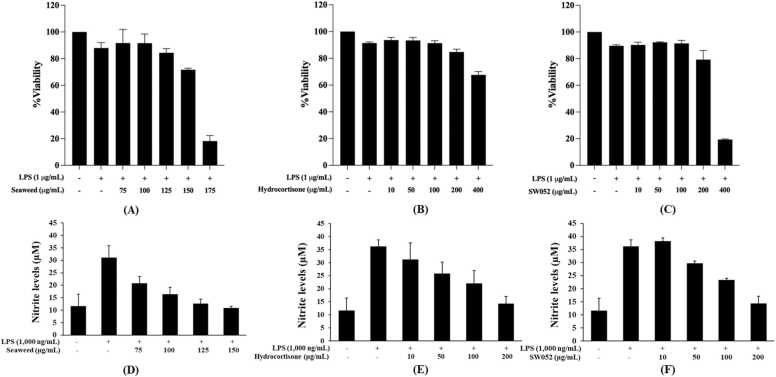
Cytotoxicity and nitric oxide inhibition of *Sargassum polycystum* extract, hydrocortisone, and 11α-hydroxyprogesterone (SW052) in RAW 264.7 macrophages. Cell viability determined by MTT assay after 24 h treatment with (A) lipophilic extract, (B) hydrocortisone and (C) 11α-hydroxyprogesterone). Nitrite levels after LPS stimulation after 24 h treatment with (D) lipophilic extract, (E) hydrocortisone and (F) 11α-hydroxyprogesterone). Values are mean ± SD (n = 3).

To determine suitable non-cytotoxic concentrations for subsequent bioassays, the cytotoxic effects of the *S. polycystum* lipophilic extract, the 11α-hydroxyprogesterone (SW052), and the reference drug hydrocortisone were evaluated in RAW 264.7 macrophages using the MTT assay. As shown in Fig. 9A-C, cell viability remained above 70 % following treatment with the lipophilic extract at concentrations of 75–150 µg/mL and with both hydrocortisone and SW052 at concentrations of 10–200 µg/mL, indicating no apparent cytotoxicity within these ranges. In contrast, exposure to higher concentrations (≥175 µg/mL for the extract and ≥400 µg/mL for hydrocortisone and SW052) resulted in a reduction of cell viability below the 70 % threshold. These higher concentrations were therefore excluded from subsequent anti-inflammatory assays. These results confirm that the *S. polycystum* lipophilic extract and SW052 are cytocompatible at the concentrations used for nitric oxide inhibition experiments.

#### 3.10.2 Anti-inflammatory activity assay

LPS stimulation (1000 ng/mL) significantly increased nitric oxide (NO) production in RAW 264.7 macrophages compared with the unstimulated control (32.16 ± 4.96 µM vs. 13.48 ± 6.10 µM; p < 0.05), confirming successful inflammatory activation. Co-treatment with the *S. polycystum* extract (75–150 µg/mL), hydrocortisone (10–200 µg/mL), or SW052 (10–200 µg/mL) markedly reduced NO production in a dose-dependent manner (Fig. 9D-F). At 100–150 µg/mL, the extract suppressed NO levels to approximately 10–12 µM, comparable to the unstimulated group (p > 0.05). Concentration response analysis revealed IC_50_ of 108.24 ± 4.64 µg/mL for the extract, 126.91 ± 3.87 µg/mL for hydrocortisone, and 144.05 ± 8.06 µg/mL for SW052 (Table 6). Statistical analysis (Tukey’s HSD, p < 0.05) indicated significant differences among treatments (Table S10). Taken together, the extract exhibited the strongest NO inhibitory effect, followed by hydrocortisone and SW052. The comparable activity of the *S. polycystum* extract to the standard corticosteroid supports the hypothesis that seaweed-derived steroidal metabolites may act as natural GR modulators, capable of attenuating macrophage mediated inflammation without cytotoxicity. The comparable inhibition of NO production by SW052 and hydrocortisone suggests that seaweed-derived steroidal metabolites may act as natural GR modulators capable of attenuating macrophage-mediated inflammation without cytotoxicity.

**Table 6:** IC_50_ values for nitric oxide inhibition in LPS-stimulated RAW 264.7 macrophages treated with the *S. polycystum* lipophilic extract, hydrocortisone (HC), and 11α-hydroxyprogesterone (SW052). Values are expressed as mean ± SD (n = 3). Different superscript letters indicate significant differences among treatments according to Tukey’s HSD test (p < 0.05).

**Samples**	**IC**_**50**_**(µg/mL)**
*S. polycystum* lipophilic extract	108.24 ± 4.64^a^
Hydrocortisone	126.91 ± 3.87^b^
11α-hydroxyprogesterone (SW052)	144.05 ± 8.60^c^

## 4 Discussion

This study establishes an integrated computational-experimental framework to explore the interaction between seaweed-derived steroidal metabolites and the GR, a central regulator of inflammatory signaling. By combining cheminformatics, network pharmacology, molecular docking, *in silico* ADMET profiling, MD simulations, and *in vitro* validation, the workflow provides a systematic approach to connect chemical molecular structure with predicted macromolecular interactions and observed cellular anti-inflammatory responses. Among the screened metabolites, 11α-hydroxyprogesterone (SW052) from *S. polycystum* was prioritized based on its structural compatibility with the GR ligand-binding domain, favorable drug-likeliness, and concentration-dependent inhibition of NO production in activated macrophages. These findings provide computational and preliminary biological evidence at the structural interaction and cellular response levels, supporting the relevance of a seaweed-derived steroidal metabolite scaffold in GR-associated anti-inflammatory research.

Cheminformatics and network-based analyses revealed that several seaweed-derived steroidal metabolites share high structural similarity (Tc ≥ 0.85) with approved steroidal anti-inflammatory drugs, particularly glucocorticoid analogues. This observation supports the hypothesis that marine secondary metabolites can recapitulate key pharmacophoric features of clinically active steroids, potentially enabling interaction with shared molecular targets such as nuclear receptors [10], [11]. Network-based analysis further identified GR (NR3C1) as a central node within a steroid-enriched functional cluster, that also included other hormone-responsive receptors such as PPARA, PPARG and ESR1. This pattern is compatible with preferential involvement of nuclear receptor-associated pathways, rather than cyclooxygenase-dependent mechanisms [12], [25].

The structural resemblance between marine sterols and endogenous corticosteroids likely reflects the biosynthetic versatility of marine macroalgae, which produce sterols and terpenoid derivatives featuring conserved steroid nuclei with hydroxylated or unsaturated side chains [11], [70]. Steroidal metabolites such as fucosterol and 11α-hydroxyprogesterone (SW052) share a C21 steroidal backbone and lipophilic framework that favor hydrophobic packing within the GR ligand-binding domain [20]. These features are predicted to support stable ligand accommodation through van der Waals and π-alkyl interactions characteristic of steroid–receptor recognition, while oxygenated functional groups may further contribute to affinity by forming hydrogen bonds. Collectively, these observations support a structure-interaction relationship, linking algal chemical diversity to macromolecular recognition processes relevant to inflammatory regulation, rather than direct functional modulation. Similar structure-guided bioactive scaffolds have been reported among diverse classes of marine-derived metabolites, showing how algal secondary metabolites can engage biological targets through distinct molecular interactions without implying shared mechanisms of functional equivalence. For example, fucoxanthin has been reported to induce apoptosis in cancer cells via mitochondrial-dependent pathways that resemble the mechanisms of certain chemotherapeutic agents [71]. Phlorotannins have been reported to inhibit α-glucosidase and protein tyrosine phosphatase 1B (PTP1B), exhibiting antidiabetic activities comparable to those of clinically used enzyme inhibitors [72]. In addition, sulfated polysaccharides from seaweed modulate immune responses through interaction with toll-like receptor and downstream cytokine signaling pathways, thereby influencing inflammation-related processes [21], [22]. These examples serve as precedents highlighting the chemical diversity and scaffold versatility of marine macroalgae. Importantly, these marine-derived scaffolds are frequently associated with favorable safety and biocompatibility profiles, supporting their potential as sustainable and structurally diverse leads for next-generation therapeutic development [10], [73].

Molecular docking was employed primarily to predict the initial binding pose of SW052 within the GR ligand-binding domain. Based on the docking results, hydrogen-bond geometry was evaluated to assess interaction quality, with donor-acceptor distances below approximately 3.5 Å identified as geometrically favorable and commonly associated with stable ligand-receptor interactions [74]. Notably, although both ligands exhibited favorable hydrogen-bond geometries, the shorter hydrogen-bond distances observed for hydrocortisone are consistent with stronger interaction strength and enhanced complex stability, in accordance with established structure-interaction principles [75]. To further characterize ligand–receptor interactions beyond static docking predictions, MD simulations were performed. The 500 ns trajectories demonstrated that the GR-SW052 complex rapidly reached equilibrium and maintained stable backbone RMSD and radius of gyration values throughout the simulation period, indicating a persistent and structurally stable binding configuration. This stability was further supported by the reproducibility of the binding orientation across triplicate trajectories and subsequent cluster analysis, confirming the robustness of the observed interaction mode. Analysis of key residues from representative MD conformations revealed that SW052 binding is predominantly stabilized by hydrophobic and van der Waals interactions with residues such as MET560, LEU563, LEU566, MET601, MET604, LEU732, TYR735, and CYS736, reflecting structural complementarity between the steroidal scaffold and the GR ligand-binding cavity. In contrast, hydrocortisone engaged a broader and more diverse set of stabilizing residues, resulting in a more extensive interaction network. This expanded interaction profile, incorporating both hydrophobic and electrostatic interactions, is consistent with its more favorable binding free energy relative to the primarily hydrophobic-driven binding observed for SW052. Notably, while both ligands maintained hydrophobic interactions with key residues such as MET560, only hydrocortisone formed H-bond with THR739, a residue previously identified as a critical H-bonding site in GR-steroid interaction [76].

To provide pharmacological context, hydrocortisone and the high-affinity synthetic glucocorticoid dexamethasone were included as benchmark ligands. Although SW052 exhibited a predicted binding affinity (−11.6 kcal/mol) that was slightly more favorable than those of hydrocortisone and dexamethasone (−11.1 kcal/mol), this modest difference falls within the inherent uncertainty of docking-based scoring functions and should therefore be interpreted as a relative trend rather than an absolute measure of binding strength. Importantly, the superior in vivo potency of dexamethasone has been attributed to deliberate structural optimizations of the steroid scaffold that enhance receptor activation efficiency and stabilize ligand-induced receptor conformations, rather than being determined solely by static binding affinity. This interpretation is consistent with previous structure–function analyses demonstrating that glucocorticoid potency reflects a combination of ligand-dependent receptor conformational states and downstream transcriptional competence [77]. In this context, the docking and simulation results indicate that SW052 adopts a binding mode more closely resembling that of endogenous glucocorticoids, supporting its classification as a naturally derived steroidal lead scaffold rather than a clinically optimized therapeutic agent.

The energetic basis of these interactions was further quantified using MM/PB(GB)SA calculations. For SW052, the total binding free energy was dominated by van der Waals interactions (Δ*E*_vdW_ = −49.82 kcal/mol), whereas the electrostatic interaction contributed to a lesser extent (Δ*E*_ele_ = −13.33 kcal/mol). This energetic profile confirms that SW052 stabilization is primarily governed by shape complementarity and hydrophobic packing, consistent with the established role of the steroidal core in directing binding orientation within the GR pocket [78]. In contrast, hydrocortisone exhibited a markedly larger electrostatic contribution (Δ*E*_ele_ = −30.89 kcal/mol), in agreement with its more extensive polar and electrostatic interaction network. Importantly, these MM/PB(GB)SA estimates reflect relative binding affinity trends rather than absolute experimental free energies [79], [80], with accuracy dependent on simulation length and conformational sampling [81], [82]. Although the PB model is theoretically more rigorous for highly polar binding environments such as the GR pocket, the GB approach provided complementary validation to ensure consistency in ligand ranking. This dual-model strategy mitigates variability associated with implicit solvent treatments, as GB models have been reported to yield comparable or, in some cases, superior performance for specific systems [83]. Consequently, MM/PBSA results were emphasized in the final analysis, as they more effectively captured the energetic distinction between the hydrophobic-driven binding of SW052 and the polar–electrostatic interaction network characteristic of hydrocortisone.Importantly, while MD simulations support the structural feasibility and stability of SW052 binding to the GR ligand-binding domain, they do not provide evidence of receptor activation or downstream transcriptional signaling. Accordingly, mechanistic conclusions regarding GR-mediated anti-inflammatory action must remain tentative. To explore potential off-target structural compatibility within the nuclear receptor family, additional docking analyses were conducted against the progesterone receptor (PR). SW052 exhibited a lower predicted binding affinity toward PR (-11.2 kcal/mol) than toward GR (-11.6 kcal/mol) within the applied docking framework. However, these docking-based observations provide only preliminary insight into receptor compatibility and cannot be interpreted as evidence of functional selectivity or hormonal activity.

Although 11α-hydroxyprogesterone has previously been reported in microbial systems as a product of progesterone biotransformation [84], its occurrence in marine macroalgae has only recently been identified through metabolomic surveys of *S. polycystum* [30]. To our knowledge, this study provides the first integrated computational and cellular evidence linking a seaweed-derived 11α-hydroxyprogesterone (SW052) scaffold to GR-related anti-inflammatory potential at structural interaction and cellular response levels. SW052 demonstrated stable GR binding in MD simulations and inhibited NO production in activated macrophages without detectable cytotoxicity. These findings are compatible with, but do not prove, involvement of GR-associated pathways, and direct validation using receptor-reporter assays or GR-responsive gene expression analysis is required to substantiate this mechanistic hypothesis.

Consistent with trends commonly observed in natural product pharmacology, the lipophilic crude extract of *S. polycystum* exhibited stronger NO-inhibitory activity than the isolated compound SW052, suggesting potential synergistic effects among co-extracted metabolites [16], [17]. *In silico* ADMET profiling further indicated that SW052 complies with Lipinski’s Rule of Five, exhibits high predicted gastrointestinal absorption, moderate lipophilicity, and low predicted systemic toxicity, supporting its feasibility as a bioactive scaffold. Nevertheless, important limitations remain, including the absence of direct evidence for GR activation and the lack of *in vivo* validation. Future studies incorporating receptor-specific functional assays and animal inflammation models will be essential to confirm efficacy, selectivity, and safety. Overall, this work highlights SW052 as a structurally feasible marine-derived steroid scaffold for further investigation.

## 5 Conclusion

This study identifies 11α-hydroxyprogesterone (SW052) from *S. polycystum* as a seaweed-derived steroidal metabolite with predicted relevance to the GR and preliminary cellular effects consistent with anti-inflammatory potential. By integrating cheminformatics, network pharmacology, molecular docking, 500 ns MD simulations, and *in vitro* screening assays, this study demonstrates computationally predicted stable binding of SW052 to the GR with binding affinity comparable to hydrocortisone, and experimental observations of NO suppression in macrophages without apparent cytotoxicity. The favorable *in silico* ADMET profile and structural resemblance to clinical corticosteroids suggest its potential as a natural lead scaffold for the development of safer steroid-like agents. Overall, these findings establish a computational and structural framework for prioritizing marine steroidal metabolites with potential GR relevance and provide a reproducible workflow for mechanism-oriented drug discovery from seaweeds. However, direct functional validation of GR signaling remains essential. Future studies should assess canonical GR-responsive genes (e.g., GILZ, FKBP5) and additional downstream markers to determine whether SW052 elicits GR-dependent transcriptional activity. Additionally, *in vivo* studies will also be required to confirm its therapeutic relevance. Nevertheless, the present work reinforces the promise of marine biodiversity as a source of bio-derived anti-inflammatory candidates.

## CRediT authorship contribution statement

**Supatchar Sermsakulwat:** Writing - original draft, Visualization, Validation, Software, Methodology, Investigation, Formal analysis, Data curation. **Phuphiphat Jaikaew:** Writing - original draft, Validation, Software, Resources, Methodology, Funding acquisition, Conceptualization. **Tiwtawat Napiroon:** Resources, Methodology, Funding acquisition. **Worawat Surarit:** Writing - original draft, Visualization, Validation, Resources, Methodology, Formal analysis. **Thrissawan Traijitt:** Resources, Methodology. **Theppanya Charoenrat:** Visualization, Validation, Resources, Methodology. **Bodee Nutho:** Writing - original draft, Visualization, Validation, Software, Resources, Methodology. **Arachaporn Thong-olran:** Visualization, Resources, Data curation. **Supenya Chittapun:** Writing - review & editing, Writing-original draft, Visualization, Validation, Supervision, Resources, Project administration, Methodology, Funding acquisition, Formal analysis, Conceptualization.

## Ethical approval

Not applicable.

## Funding

This research was supported by the Thailand Science Research and Innovation (TSRI) Fundamental Fund, fiscal year 2025, 10.13039/501100005790Thammasat University (Contract No. TUFF 27/2568).

## Declaration of Competing Interest

The authors declare no competing financial interests or personal relationships that could have appeared to influence the work reported in this paper.
