# Supplementary material for: Computational and experimental insights into the interaction of the seaweed-derived steroidal metabolite 11α-hydroxyprogesterone with the glucocorticoid receptor
Source: Comput Struct Biotechnol J. 2025 Dec 30;31:202–20. doi: 10.1016/j.csbj.2025.12.028 (PMC12809411; doi:10.1016/j.csbj.2025.12.028)
Supplement: Table S1 — Supplementary material [file mmc1.docx]

**Fig. S1.** Additional molecular dynamics simulations data from two independent MD replicates.

**
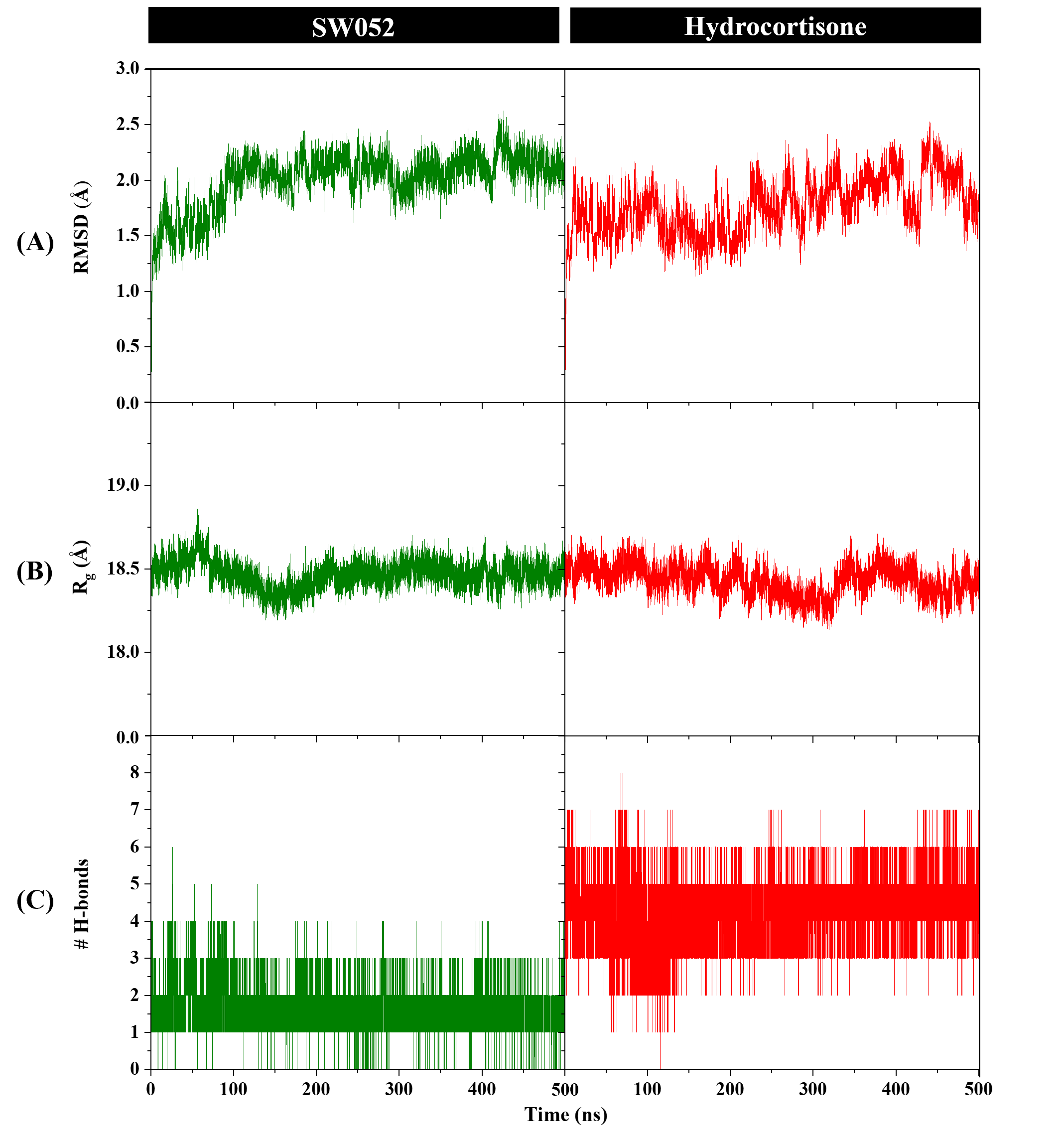
**

**Fig. S1 (1).** Time evolution of (A) RMSD, (B) radius of gyration (Rg), and (C) hydrogen bonding profiles for GR complexes with SW052 and hydrocortisone during 500 ns MD simulations from the second independent trajectory. **(second replication)**


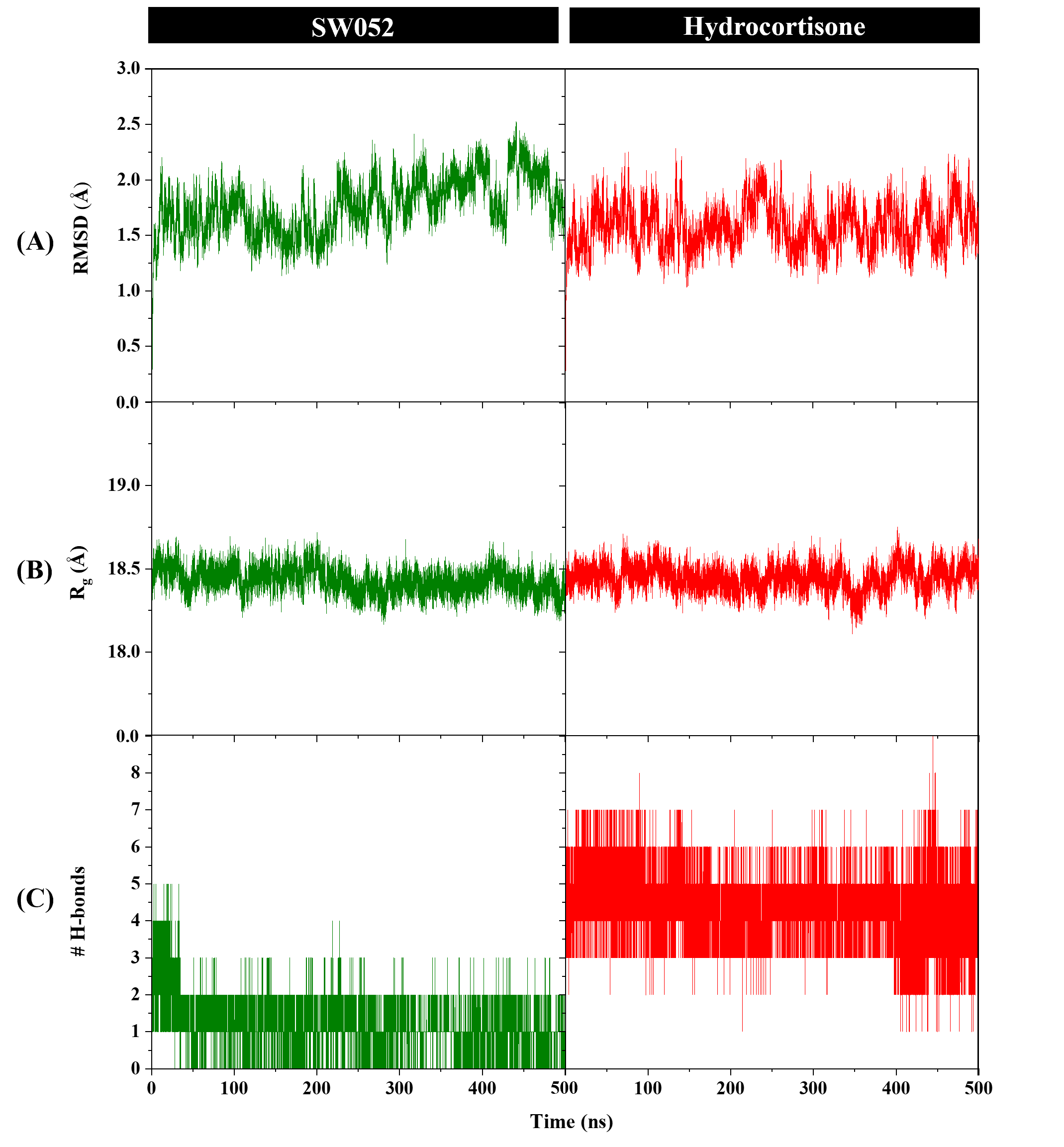


**Fig. S1 (2).** Time evolution of (A) RMSD, (B) radius of gyration (Rg), and (C) hydrogen bonding profiles for GR complexes with SW052 and hydrocortisone during 500 ns MD simulations from the third independent trajectory. (thrid replication)
