# Supplementary material for: Computational and experimental insights into the interaction of the seaweed-derived steroidal metabolite 11α-hydroxyprogesterone with the glucocorticoid receptor
Source: Comput Struct Biotechnol J. 2025 Dec 30;31:202–20. doi: 10.1016/j.csbj.2025.12.028 (PMC12809411; doi:10.1016/j.csbj.2025.12.028)
Supplement: Table S12 — Supplementary material [file mmc12.docx]

**Table S10**. One-way ANOVA of IC_50_ values of NO inhibition, including F-statistics, degrees of freedom, and exact p-values (n=3).

| **Source of variation** | **Sum of squares** | **df** | **Mean square** | **F** | **p-value** |
| --- | --- | --- | --- | --- | --- |
| Between groups | 2226.45 | 2 | 1113.23 | 34.61 | < 0.001 |
| Within groups | 257.32 | 8 | 32.17 |  |  |
| Total | 2483.77 | 10 |  |  |  |
