# Supplementary material for: Computational and experimental insights into the interaction of the seaweed-derived steroidal metabolite 11α-hydroxyprogesterone with the glucocorticoid receptor
Source: Comput Struct Biotechnol J. 2025 Dec 30;31:202–20. doi: 10.1016/j.csbj.2025.12.028 (PMC12809411; doi:10.1016/j.csbj.2025.12.028)
Supplement: Table S2 — Supplementary material [file mmc2.docx]

**Fig. S2.** Cluster analysis of GR–ligand binding poses (protein backbone and protein backbone with ligand) derived from the final 50 ns of triplicate MD trajectories.


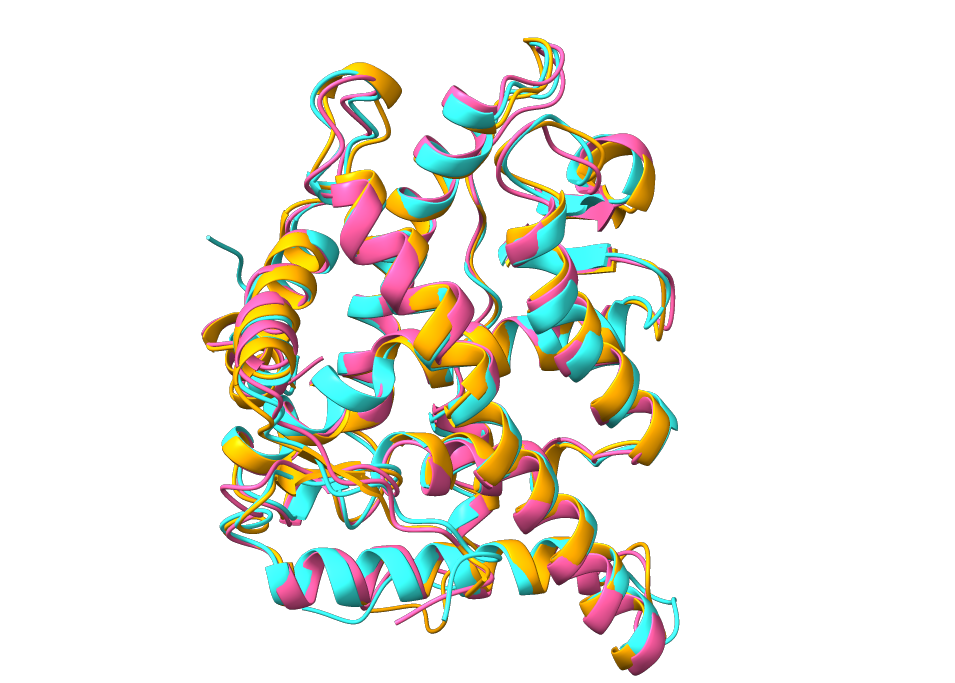


**Fig. S2 (1).** Superposition of representative **GR–SW052 binding poses** **(protein backbone)** derived from three independent MD trajectories during the final 50 ns after equilibration.


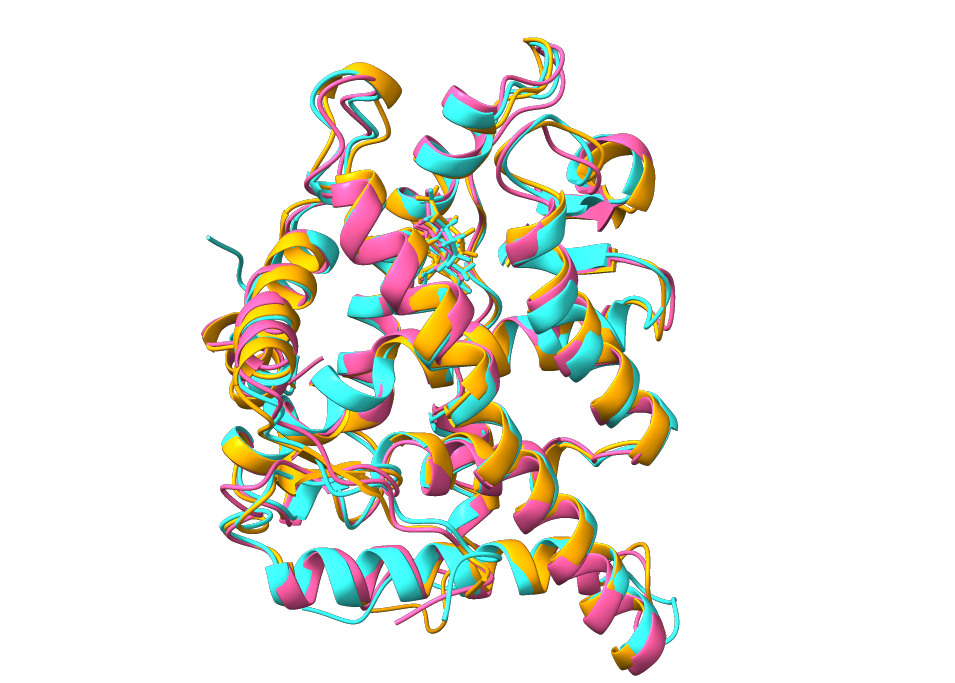


**Fig. S2 (2).** Superposition of representative **GR–SW052 binding poses (including ligand-bound conformations)** derived from three independent MD trajectories during the final 50 ns after equilibration.


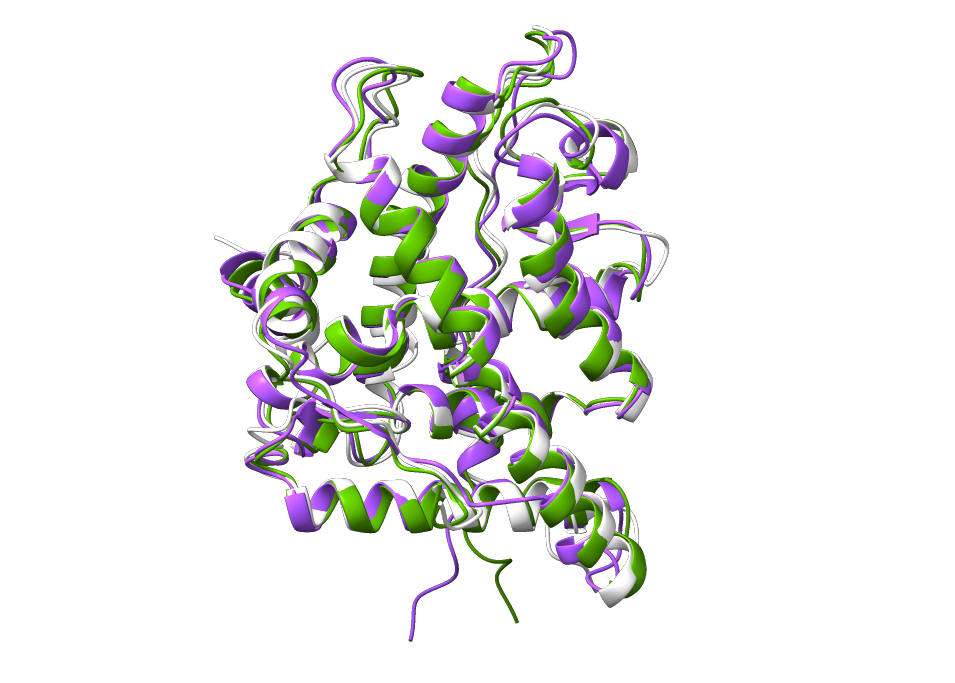


**Fig. S2 (3).** Superposition of representative **GR–hydrocortisone binding poses (protein backbone)** derived from three independent MD trajectories during the final 50 ns after equilibration.
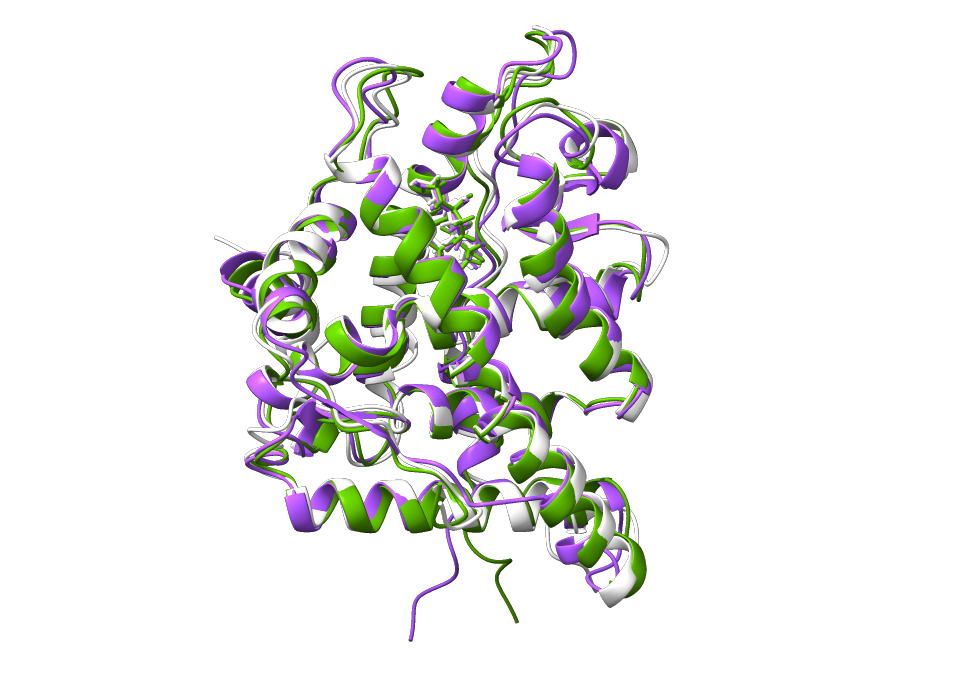


**Fig. S2 (4).** Superposition of representative **GR–hydrocortisone binding poses (including ligand-bound conformations)** derived from three independent MD trajectories during the final 50 ns after equilibration.
