# Supplementary material for: Computational and experimental insights into the interaction of the seaweed-derived steroidal metabolite 11α-hydroxyprogesterone with the glucocorticoid receptor
Source: Comput Struct Biotechnol J. 2025 Dec 30;31:202–20. doi: 10.1016/j.csbj.2025.12.028 (PMC12809411; doi:10.1016/j.csbj.2025.12.028)
Supplement: Table S3 — Supplementary material [file mmc3.docx]

**Table S1. Structural similarities with fingerprint analysis between bioactive compounds from seaweed and drugs in DrugBank (Tc ≥ 0.85)**

| **No.** | **Accession Number** | **Compound** | **Drug ID** | **Drug** | **MACCS** | **Daylight** | **Morgan** | **ECFP6** |
| --- | --- | --- | --- | --- | --- | --- | --- | --- |
| **1** | SW001 | Methyl 13,16-docosadienoate | DB01631 | Methyl nonanoate | 0.95 | - | - | - |
| **2** | SW003 | Cyclopropane butanoic acid, 2-[2-[[2-[(2-pentylcyclopropyl) methyl] cyclopropyl] methyl] cyclopropyl] methyl]-, methyl ester | DB01631 | Methyl nonanoate | 0.86 | - | - | - |
| **3** | SW005 | Propanoic acid, 2-(3-acetoxy-4,4,14-trimethylandrost-8-en-17-yl)- | DB00253 | Medrysone | 0.88 | - | - | - |
|  |  |  | DB00351 | Megestrol acetate | 0.9 | - | - | - |
|  |  |  | DB00603 | Medroxyprogesterone acetate | 0.9 | - | - | - |
|  |  |  | DB00823 | Ethynodiol diacetate | 0.9 | - | - | - |
|  |  |  | DB01134 | Desoxycorticosterone pivalate | 0.86 | - | - | - |
|  |  |  | DB01420 | Testosterone propionate | 0.85 | - | - | - |
|  |  |  | DB02329 | Carbenoxolone | 0.88 | - | - | - |
|  |  |  | DB02703 | Fusidic acid | 0.88 | - | - | - |
|  |  |  | DB06789 | Hydroxyprogesterone caproate | 0.86 | - | - | - |
|  |  |  | DB08547 | Progesterone-11α-ol-hemisuccinate | 0.88 | - | - | - |
|  |  |  | DB13943 | Testosterone cypionate | 0.85 | - | - | - |
|  |  |  | DB13944 | Testosterone enanthate | 0.85 | - | - | - |
|  |  |  | DB13946 | Testosterone undecanoate | 0.85 | - | - | - |
|  |  |  | DB13981 | Nomegestrol acetate | 0.9 | - | - | - |
|  |  |  | DB14583 | Segesterone acetate | 0.88 | - | - | - |
|  |  |  | DB14659 | Melengestrol acetate | 0.88 | - | - | - |
| **4** | SW007 | Tridecanoic acid, 12-methyl-, methyl ester | DB01631 | Methyl nonanoate | 0.86 | 0.89 | - | - |
| **5** | SW008 | Methyl tetradecanoate | DB01631 | Methyl nonanoate | 1 | 1 | 1 | 1 |
| **6** | SW010 | 9α-Fluoro-17α-methyl-4-androsten-3α, 6β,11β,17β-tetra-ol | DB00324 | Fluorometholone | 0.93 | - | - | - |
|  |  |  | DB00764 | Mometasone | 0.85 | - | - | - |
|  |  |  | DB01185 | Fluoxymesterone | 0.95 | - | - | - |
|  |  |  | DB11750 | Clobetasol | 0.87 | - | - | - |
| **7** | SW011 | Methyl 13-methyltetradecanoate | DB01631 | Methyl nonanoate | 0.86 | 0.89 | - | - |
| **8** | SW012 | Tetradecanoic acid, 12-methyl-, methyl ester | DB01631 | Methyl nonanoate | 0.95 | - | - | - |
| **9** | SW013 | Pentadecanoic acid, methyl ester | DB01631 | Methyl nonanoate | 1 | 1 | 1 | 1 |
| **10** | SW014 | 2,3-Dimethoxy-5-methyl-6-dekaisoprenyl-chinon | DB08689 | Ubiquinone Q1 | - | 0.99 | - | - |
|  |  |  | DB08690 | Ubiquinone Q2 | 1 | 1 | 0.97 | 0.92 |
|  |  |  | DB09270 | Ubidecarenone | 1 | 1 | 1 | 1 |
| **11** | SW016 | 13-Heptadecyn-1-ol | DB06894 | 1-Dodecanol | 0.86 | - | - | - |
| **12** | SW017 | cis-10-Nonadecenoic acid | DB00132 | α-Linolenic acid | 0.9 | 0.85 | - | - |
|  |  |  | DB00154 | Dihomo-gamma-linolenic acid | 1 | 0.85 | 0.88 | - |
|  |  |  | DB00159 | Icosapent | 0.9 | - | - | - |
|  |  |  | DB02448 | N-Tridecanoic Acid | 0.95 | - | - | - |
|  |  |  | DB02938 | Heptanoic acid | 0.95 | - | - | - |
|  |  |  | DB02955 | Ricinoleic acid | 0.88 | - | - | - |
|  |  |  | DB03017 | Lauric acid | 0.95 | - | - | - |
|  |  |  | DB03193 | Stearic acid | 0.95 | - | - | - |
|  |  |  | DB03600 | Capric acid | 0.95 | - | - | - |
|  |  |  | DB03756 | Doconexent | 0.86 | - | - | - |
|  |  |  | DB03796 | Palmitic Acid | 0.95 | - | - | - |
|  |  |  | DB04224 | Oleic Acid | 1 | 1 | 1 | 1 |
|  |  |  | DB04519 | Caprylic acid | 0.95 | - | - | - |
|  |  |  | DB04557 | Arachidonic Acid | 1 | - | - | - |
|  |  |  | DB04801 | cis-Vaccenic acid | 1 | 1 | 1 | 0.94 |
|  |  |  | DB06689 | Ethanolamine oleate | - | 0.94 | - | 0.85 |
|  |  |  | DB06926 | (9Z,11E,13S)-13-hydroxyoctadeca-9,11-dienoic acid | 0.88 | - | - | - |
|  |  |  | DB07302 | 9(S)-HODE | 0.88 | - | - | - |
|  |  |  | DB08231 | Myristic acid | 0.95 | - | - | - |
| **13** | SW018 | 9-Hexadecenoic acid, methyl ester, (Z)- | DB01631 | Methyl nonanoate | 0.95 | - | - | - |
| **14** | SW019 | Hexadecanoic acid, methyl ester | DB01631 | Methyl nonanoate | 1 | 1 | 1 | 1 |
| **15** | SW021 | n-Hexadecanoic acid | DB00132 | α-Linolenic acid | 0.86 | - | - | - |
|  |  |  | DB00154 | Dihomo-gamma-linolenic acid | 0.95 | - | - | - |
|  |  |  | DB00159 | Icosapent | 0.86 | - | - | - |
|  |  |  | DB00548 | Azelaic acid | - | 1 | - | - |
|  |  |  | DB02448 | N-Tridecanoic Acid | 1 | 1 | 1 | 1 |
|  |  |  | DB02938 | Heptanoic acid | 1 | 0.96 | 1 | - |
|  |  |  | DB03017 | Lauric acid | 1 | 1 | 1 | 1 |
|  |  |  | DB03193 | Stearic acid | 1 | 1 | 1 | 1 |
|  |  |  | DB03600 | Capric acid | 1 | 1 | 1 | 1 |
|  |  |  | DB03704 | 12-Hydroxydodecanoic Acid | - | 1 | - | - |
|  |  |  | DB03796 | Palmitic Acid | 1 | 1 | 1 | 1 |
|  |  |  | DB04224 | Oleic Acid | 0.95 | - | - | - |
|  |  |  | DB04519 | Caprylic acid | 1 | 1 | 1 | 1 |
|  |  |  | DB04557 | Arachidonic Acid | 0.95 | - | - | - |
|  |  |  | DB04801 | cis-Vaccenic acid | 0.95 | - | - | - |
|  |  |  | DB05343 | Arundic acid | 0.86 | - | - | - |
|  |  |  | DB07645 | Sebacic acid | - | 1 | - | - |
|  |  |  | DB08231 | Myristic acid | 1 | 1 | 1 | 1 |
| **16** | SW022 | Estra-1,3,5(10)-trien-17β-ol | DB00783 | Estradiol | 0.91 | - | - | - |
|  |  |  | DB00977 | Ethinylestradiol | 0.88 | - | - | - |
|  |  |  | DB04575 | Quinestrol | 0.88 | - | - | - |
|  |  |  | DB06871 | 17-Methyl-17α -dihydroequilenin | 0.85 | - | - | - |
| **17** | SW023 | Oleic Acid | DB00132 | α-Linolenic acid | 0.9 | 0.85 | - | - |
|  |  |  | DB00154 | Dihomo-gamma-linolenic acid | 1 | - | - | - |
|  |  |  | DB00154 | Dihomo-gamma-linolenic acid | - | 0.85 | 0.88 | - |
|  |  |  | DB00159 | Icosapent | 0.9 | - | - | - |
|  |  |  | DB02448 | N-Tridecanoic Acid | 0.95 | - | - | - |
|  |  |  | DB02938 | Heptanoic acid | 0.95 | - | - | - |
|  |  |  | DB02955 | Ricinoleic acid | 0.88 | - | - | - |
|  |  |  | DB03017 | Lauric acid | 0.95 | - | - | - |
|  |  |  | DB03193 | Stearic acid | 0.95 | - | - | - |
|  |  |  | DB03600 | Capric acid | 0.95 | - | - | - |
|  |  |  | DB03756 | Doconexent | 0.86 | - | - | - |
|  |  |  | DB03796 | Palmitic Acid | 0.95 | - | - | - |
|  |  |  | DB04224 | Oleic Acid | 1 | 1 | 1 | 1 |
|  |  |  | DB04519 | Caprylic acid | 0.95 | - | - | - |
|  |  |  | DB04557 | Arachidonic Acid | 1 | - | - | - |
|  |  |  | DB04801 | cis-Vaccenic acid | 1 | 1 | 1 | 0.94 |
|  |  |  | DB06689 | Ethanolamine oleate | - | 0.94 | - | 0.85 |
|  |  |  | DB06926 | (9Z,11E,13S)-13-hydroxyoctadeca-9,11-dienoic acid | 0.88 | - | - | - |
|  |  |  | DB07302 | 9(S)-HODE | 0.88 | - | - | - |
|  |  |  | DB08231 | Myristic acid | 0.95 | - | - | - |
| **18** | SW024 | Ethanol, 2-(9-octadecenyloxy)-, (Z)- | DB02249 | 2-Ethoxyethanol | 0.88 | - | - | - |
|  |  |  | DB03556 | 2-(2-{2-[2-(2-{2-[2-(2-Ethoxy-Ethoxy)-Ethoxy]-Ethoxy}-Ethoxy)-Ethoxy]-Ethoxy}-Ethoxy)-Ethanol, Polyethyleneglycol Peg400 | 0.85 | - | - | - |
|  |  |  | DB04233 | (Hydroxyethyloxy)Tri(Ethyloxy)Octane | 0.89 | - | - | - |
|  |  |  | DB07344 | 3,6,9,12,15-PENTAOXAHEPTADECAN-1-OL | 0.85 | - | - | - |
|  |  |  | DB08249 | 3,6,9,12,15-PENTAOXATRICOSAN-1-OL | 0.89 | - | - | - |
| **19** | SW025 | Ursodeoxycholic acid | DB00858 | Drostanolone | 0.86 | - | - | - |
|  |  |  | DB01586 | Ursodeoxycholic acid | 1 | - | 1 | 1 |
|  |  |  | DB02659 | Cholic Acid | 1 | - | - | - |
|  |  |  | DB02703 | Fusidic acid | 0.86 | - | - | - |
|  |  |  | DB03619 | Deoxycholic acid | 1 | - | - | - |
|  |  |  | DB05087 | Ganaxolone | 0.86 | - | - | - |
|  |  |  | DB05990 | Obeticholic acid | 0.97 | - | - | - |
|  |  |  | DB06777 | Chenodeoxycholic acid | 1 | - | 1 | 1 |
|  |  |  | DB08510 | 5α -Pregnane-3β-ol-hemisuccinate | 0.89 | - | - | - |
|  |  |  | DB11859 | Brexanolone | 0.86 | - | - | - |
| **20** | SW026 | 9,10-Secocholesta-5,7,10(19)-triene-3,24,25-triol, (3β,5Z,7E)- | DB00136 | Calcitriol | 0.9 | - | - | - |
|  |  |  | DB00146 | Calcifediol | 0.93 | - | - | - |
|  |  |  | DB00169 | Cholecalciferol | 0.85 | - | - | - |
|  |  |  | DB01436 | Alfacalcidol | 0.86 | - | - | - |
| **21** | SW027 | 9,12-Octadecadienoic acid (Z,Z)-, methyl ester | DB01631 | Methyl nonanoate | 0.95 | - | - | - |
| **22** | SW028 | 9-Octadecenoic acid, methyl ester, (E)- | DB01631 | Methyl nonanoate | 0.95 | - | - | - |
| **23** | SW029 | 12-Octadecenoic acid, methyl ester | DB01631 | Methyl nonanoate | 0.95 | - | - | - |
| **24** | SW030 | Heptadecanoic acid, 16-methyl-, methyl ester | DB01631 | Methyl nonanoate | 0.86 | 0.89 | - | - |
| **25** | SW031 | 5,8,11,14-Eicosatetraenoic acid, methyl ester, (all-Z)- | DB01631 | Methyl nonanoate | 0.95 | - | - | - |
| **26** | SW032 | Docosahexaenoic acid, 1,2,3-propanetriyl ester | DB11677 | Triheptanoin | 0.9 | - | - | - |
| **27** | SW033 | 7,10,13-Eicosatrienoic acid, methyl ester | DB01631 | Methyl nonanoate | 0.95 | - | - | - |
| **28** | SW035 | Ethyl iso-allocholate | DB01586 | Ursodeoxycholic acid | 0.9 | - | - | - |
|  |  |  | DB02659 | Cholic Acid | 0.9 | - | - | - |
|  |  |  | DB03619 | Deoxycholic acid | 0.9 | - | - | - |
|  |  |  | DB05990 | Obeticholic acid | 0.93 | - | - | - |
|  |  |  | DB06777 | Chenodeoxycholic acid | 0.9 | - | - | - |
|  |  |  | DB08510 | 5α -Pregnane-3β-ol-hemisuccinate | 0.85 | - | - | - |
| **29** | SW036 | 1-Heptatriacotanol | DB06894 | 1-Dodecanol | 1 | 1 | 1 | 1 |
| **30** | SW038 | 11-Eicosenoic acid, methyl ester | DB01631 | Methyl nonanoate | 0.95 | - | - | - |
| **31** | SW043 | 13-Docosenoic acid, methyl ester | DB01631 | Methyl nonanoate | 0.95 | - | - | - |
| **32** | SW044 | Hexadecanoic acid, 1-(hydroxymethyl)-1,2-ethanediyl ester | DB07416 | (2S)-2-(BUTYRYLOXY)-3-HYDROXYPROPYL NONANOATE | 1 | - | 0.94 | 0.87 |
|  |  |  | DB11677 | Triheptanoin | 0.87 | 0.96 | - | - |
| **33** | SW048 | (22S)-6α,11β,21-Trihydroxy-16α,17α-propylmethylenedioxypregna-1,4-diene-3,20-dione | DB00180 | Flunisolide | 0.85 | - | - | - |
|  |  |  | DB00288 | Amcinonide | 0.85 | - | - | - |
|  |  |  | DB00511 | Acetyldigitoxin | 0.85 | - | - | - |
|  |  |  | DB00591 | Fluocinolone acetonide | 0.85 | - | - | - |
|  |  |  | DB00846 | Flurandrenolide | 0.89 | - | - | - |
|  |  |  | DB00959 | Methylprednisolone | 0.85 | - | - | - |
|  |  |  | DB01222 | Budesonide | 1 | - | - | - |
|  |  |  | DB01260 | Desonide | 0.96 | - | - | - |
|  |  |  | DB01410 | Ciclesonide | 0.89 | - | - | - |
|  |  |  | DB02169 | 9,10-Deepithio-9,10-Didehydroacanthifolicin | 0.85 | - | - | - |
|  |  |  | DB12499 | Clascoterone Methylprednisone | 0.86 | - | - | - |
|  |  |  | DB12952 | Methylprednisone | 0.85 | - | - | - |
|  |  |  | DB14538 | Hydrocortisone aceponate | 0.86 | - | - | - |
|  |  |  | DB14539 | Hydrocortisone acetate | 0.85 | - | - | - |
|  |  |  | DB14540 | Hydrocortisone butyrate | 0.9 | - | - | - |
|  |  |  | DB14543 | Hydrocortisone probutate | 0.86 | - | - | - |
|  |  |  | DB14544 | Hydrocortisone valerate | 0.9 | - | - | - |
|  |  |  | DB14644 | Methylprednisolone hemisuccinate | 0.85 | - | - | - |
|  |  |  | DB15566 | Prednisolone acetate | 0.85 | - | - | - |
| **34** | SW050 | Cholest-5-en-3-one | DB00378 | Dydrogesterone | 0.88 | - | - | - |
|  |  |  | DB00396 | Progesterone | 0.88 | - | - | - |
|  |  |  | DB01456 | 5-androstenedione | 0.85 | - | - | - |
|  |  |  | DB01536 | Androstenedione | 0.85 | - | - | - |
|  |  |  | DB04540 | Cholesterol | 0.86 | - | - | - |
|  |  |  | DB09124 | Medrogestone | 0.88 | - | - | - |
|  |  |  | DB13602 | Promegestone | 0.88 | - | - | - |
|  |  |  | DB13857 | Demegestone | 0.88 | - | - | - |
| **35** | SW051 | Stigmasta-5,24(28)-dien-3-ol, (3β,24Z)- | DB00146 | Calcifediol | 0.87 | - | - | - |
|  |  |  | DB00153 | Ergocalciferol | 0.91 | - | - | - |
|  |  |  | DB00169 | Cholecalciferol | 0.94 | - | - | - |
|  |  |  | DB01070 | Dihydrotachysterol | 0.94 | - | - | - |
|  |  |  | DB01431 | Allylestrenol | 0.86 | - | - | - |
|  |  |  | DB01564 | Calusterone | 0.86 | - | - | - |
|  |  |  | DB01708 | Prasterone | 0.86 | - | - | - |
|  |  |  | DB02789 | Pregnenolone | 0.89 | - | - | - |
|  |  |  | DB03696 | Lanosterol | 0.97 | - | - | - |
|  |  |  | DB04540 | Cholesterol | 1 | - | - | - |
|  |  |  | DB06710 | Methyltestosterone | 0.86 | - | - | - |
|  |  |  | DB11429 | Mibolerone | 0.86 | - | - | - |
| **36** | SW052 | 11-Hydroxyprogesterone | DB00253 | Medrysone | 1 | - | - | - |
|  |  |  | DB00378 | Dydrogesterone | 0.86 | - | - | - |
|  |  |  | DB00396 | Progesterone | 0.86 | - | - | - |
|  |  |  | DB00624 | Testosterone | 0.89 | - | - | - |
|  |  |  | DB00896 | Rimexolone | 0.95 | - | - | - |
|  |  |  | DB01541 | Boldenone | 0.89 | - | - | - |
|  |  |  | DB01564 | Calusterone | 0.92 | - | - | - |
|  |  |  | DB01569 | Formebolone | 0.9 | - | - | - |
|  |  |  | DB01708 | Prasterone | 0.86 | - | - | - |
|  |  |  | DB02789 | Pregnenolone | 0.89 | - | - | - |
|  |  |  | DB02998 | Metribolone | 0.89 | - | - | - |
|  |  |  | DB05212 | HE3286 | 0.85 | - | - | - |
|  |  |  | DB06412 | Oxymetholone | 0.87 | - | - | - |
|  |  |  | DB06710 | Methyltestosterone | 0.92 | - | - | - |
|  |  |  | DB07768 | Epitestosterone | 0.89 | - | - | - |
|  |  |  | DB08547 | Progesterone-11α -ol-hemisuccinate | 0.85 | - | - | - |
|  |  |  | DB08905 | Formestane | 0.89 | - | - | - |
|  |  |  | DB09070 | Tibolone | 0.87 | - | - | - |
|  |  |  | DB09124 | Medrogestone | 0.86 | - | - | - |
|  |  |  | DB11429 | Mibolerone | 0.92 | - | - | - |
|  |  |  | DB11529 | Melengestrol | 0.9 | - | - | - |
|  |  |  | DB11636 | Nomegestrol | 0.95 | - | - | - |
|  |  |  | DB13528 | Chlormadinone | 0.86 | - | - | - |
|  |  |  | DB13857 | Demegestone | 0.86 | - | - | - |
|  |  |  | DB14570 | Hydroxyprogesterone | 0.95 | - | - | - |
| **37** | SW053 | Stigmasterol | DB00146 | Calcifediol | 0.85 | - | - | - |
|  |  |  | DB00153 | Ergocalciferol | 0.89 | - | - | - |
|  |  |  | DB00169 | Cholecalciferol | 0.92 | - | - | - |
|  |  |  | DB01070 | Dihydrotachysterol | 0.91 | - | - | - |
|  |  |  | DB02789 | Pregnenolone | 0.86 | - | - | - |
|  |  |  | DB03696 | Lanosterol | 0.94 | - | - | - |
|  |  |  | DB04540 | Cholesterol | 0.97 | - | - | - |
| **38** | SW054 | Astaxanthin | DB06543 | Astaxanthin | 1 | 1 | 1 | 1 |
| **39** | SW055 | Erucic acid | DB00132 | α-Linolenic acid | 0.9 | 0.85 | - | - |
|  |  |  | DB00154 | Dihomo-gamma-linolenic acid | 1 | - | 0.88 | - |
|  |  |  | DB00154 | Dihomo-gamma-linolenic acid | - | 0.85 | - | - |
|  |  |  | DB00159 | Icosapent | 0.9 | - | - | - |
|  |  |  | DB02448 | N-Tridecanoic Acid | 0.95 | - | - | - |
|  |  |  | DB02938 | Heptanoic acid | 0.95 | - | - | - |
|  |  |  | DB02955 | Ricinoleic acid | 0.88 | - | - | - |
|  |  |  | DB03017 | Lauric acid | 0.95 | - | - | - |
|  |  |  | DB03193 | Stearic acid | 0.95 | - | - | - |
|  |  |  | DB03600 | Capric acid | 0.95 | - | - | - |
|  |  |  | DB03756 | Doconexent | 0.86 | - | - | - |
|  |  |  | DB03796 | Palmitic Acid | 0.95 | - | - | - |
|  |  |  | DB04224 | Oleic Acid | 1 | 1 | 1 | 1 |
|  |  |  | DB04519 | Caprylic acid | 0.95 | - | - | - |
|  |  |  | DB04557 | Arachidonic Acid | 1 | - | - | - |
|  |  |  | DB04801 | cis-Vaccenic acid | 1 | 1 | 1 | 0.94 |
|  |  |  | DB06689 | Ethanolamine oleate | - | 0.94 | - | 0.85 |
|  |  |  | DB06926 | (9Z,11E,13S)-13-hydroxyoctadeca-9,11-dienoic acid | 0.88 | - | - | - |
|  |  |  | DB07302 | 9(S)-HODE | 0.88 | - | - | - |
|  |  |  | DB08231 | Myristic acid | 0.95 | - | - | - |
| **40** | SW058 | Azuleno[4,5-b]furan-2,9-dione, decahydro-6a-hydroxy-6,9a-dimethyl-3-methylene-, [3aS-(3aα,6β,6aα,9aβ,9bα)]- | DB07815 | Gibberellin A4 | 0.89 | - | - | - |
| **41** | SW059 | 2-[4-methyl-6-(2,6,6-trimethylcyclohex-1-enyl)hexa-1,3,5-trienyl]cyclohex-1-en-1-carboxaldehyde | DB06755 | β-Carotene | 0.86 | - | - | - |
| **42** | SW060 | Fenretinide | DB05076 | Fenretinide | 1 | - | 1 | 1 |
| **43** | SW064 | 10,12,14-Nonacosatriynoic acid | DB00154 | Dihomo-gamma-linolenic acid | 0.91 | - | - | - |
|  |  |  | DB02448 | N-Tridecanoic Acid | 0.95 | - | - | - |
|  |  |  | DB02938 | Heptanoic acid | 0.95 | - | - | - |
|  |  |  | DB03017 | Lauric acid | 0.95 | - | - | - |
|  |  |  | DB03193 | Stearic acid | 0.95 | - | - | - |
|  |  |  | DB03600 | Capric acid | 0.95 | - | - | - |
|  |  |  | DB03796 | Palmitic Acid | 0.95 | - | - | - |
|  |  |  | DB04224 | Oleic Acid | 0.91 | - | - | - |
|  |  |  | DB04519 | Caprylic acid | 0.95 | - | - | - |
|  |  |  | DB04557 | Arachidonic Acid | 0.91 | - | - | - |
|  |  |  | DB04801 | cis-Vaccenic acid | 0.91 | - | - | - |
|  |  |  | DB08231 | Myristic acid | 0.95 | - | - | - |
| **44** | SW067 | 11-Hexadecenoic acid, methyl ester | DB01631 | Methyl nonanoate | 0.95 | - | - | - |
| **45** | SW071 | 16-Octadecenoic acid, methyl ester | DB01631 | Methyl nonanoate | 0.85 | - | - | - |
| **46** | SW072 | Methyl oleate | DB01631 | Methyl nonanoate | 0.95 | - | - | - |
| **47** | SW074 | Heptadecanoic acid, 9-methyl-, methyl ester | DB01631 | Methyl nonanoate | 0.95 | - | - | - |
| **48** | SW075 | Methyl stearate | DB01631 | Methyl nonanoate | 1 | 1 | 1 | 1 |
| **49** | SW077 | cholestan-3-ol, 2-methylene-, (3β,5α) | DB00136 | Calcitriol | 0.88 | - | - | - |
|  |  |  | DB00146 | Calcifediol | 0.95 | - | - | - |
|  |  |  | DB00153 | Ergocalciferol | 0.94 | - | - | - |
|  |  |  | DB00169 | Cholecalciferol | 0.97 | - | - | - |
|  |  |  | DB01070 | Dihydrotachysterol | 0.91 | - | - | - |
|  |  |  | DB01431 | Allylestrenol | 0.89 | - | - | - |
|  |  |  | DB01436 | Alfacalcidol | 0.87 | - | - | - |
|  |  |  | DB03696 | Lanosterol | 0.94 | - | - | - |
|  |  |  | DB04540 | Cholesterol | 0.92 | - | - | - |
|  |  |  | DB06410 | Doxercalciferol | 0.85 | - | - | - |
| **50** | SW080 | [9-(Acetyloxy)-4a,7b,9a-trihydroxy-1,1,6,8-tetramethyl-5-oxo-1a,1b,4,4a,5,7a,7b,8,9,9a-decahydro-1H-cyclopropa[3,4]benzo[1,2-E]azulen-3-yl]methyl acetate | DB04376 | 13-Acetylphorbol | 0.93 | - | - | - |
|  |  |  | DB05013 | Ingenol mebutate | 0.85 | - | - | - |
| **51** | SW084 | Cholestane-3,5-diol, 5-acetate, (3β,5α)- | DB01586 | Ursodeoxycholic acid | 0.87 | - | - | - |
|  |  |  | DB02659 | Cholic Acid | 0.87 | - | - | - |
|  |  |  | DB03619 | Deoxycholic acid | 0.87 | - | - | - |
|  |  |  | DB05990 | Obeticholic acid | 0.85 | - | - | - |
|  |  |  | DB06777 | Chenodeoxycholic acid | 0.87 | - | - | - |
|  |  |  | DB08510 | 5α -pregnane-3β-ol-hemisuccinate | 0.87 | - | - | - |
| **52** | SW087 | 3-Desoxo-3,16-dihydroxy-12-desoxyphorbol 3,13,16,20-tetraacetate | DB04376 | 13-Acetylphorbol | 0.85 | - | - | - |
| **53** | SW088 | Olean-12-ene-3,15,16,21,22,28-hexol, (3β,15α,16α,21β,22α)- | DB00635 | Prednisone | 0.88 | - | - | - |
|  |  |  | DB00741 | Hydrocortisone | 0.88 | - | - | - |
|  |  |  | DB00860 | Prednisolone | 0.88 | - | - | - |
|  |  |  | DB00959 | Methylprednisolone | 0.9 | - | - | - |
|  |  |  | DB05295 | Eldecalcitol | 0.88 | - | - | - |
|  |  |  | DB09383 | Meprednisone | 0.88 | - | - | - |
|  |  |  | DB12952 | Methylprednisone | 0.9 | - | - | - |
|  |  |  | DB13208 | Prednylidene | 0.86 | - | - | - |
|  |  |  | DB14681 | Cortisone | 0.88 | - | - | - |
| **54** | SW089 | 24-Ethylcholest-5-en-3β -ol | DB00146 | Calcifediol | 0.85 | - | - | - |
|  |  |  | DB00153 | Ergocalciferol | 0.89 | - | - | - |
|  |  |  | DB00169 | Cholecalciferol | 0.92 | - | - | - |
|  |  |  | DB01070 | Dihydrotachysterol | 0.91 | - | - | - |
|  |  |  | DB02789 | Pregnenolone | 0.86 | - | - | - |
|  |  |  | DB03696 | Lanosterol | 0.94 | - | - | - |
|  |  |  | DB04540 | Cholesterol | 0.97 | - | - | - |
| **55** | SW090 | 9,19-Cyclolanostane-3,7-diol | DB00146 | Calcifediol | 0.85 | - | - | - |
|  |  |  | DB03882 | 5α-Androstane-3β,17β-Diol | 0.86 | - | - | - |
|  |  |  | DB03926 | Hydrocortisone | 0.86 | - | - | - |
| **56** | SW091 | Glycodeoxycholic acid | DB02123 | Glycochenodeoxycholic Acid | 1 | 0.93 | - | - |
|  |  |  | DB02691 | Glycocholic acid | 0.96 | - | - | - |
| **57** | SW092 | Triarachidin | DB07416 | (2S)-2-(BUTYRYLOXY)-3-HYDROXYPROPYL NONANOATE | 0.87 | - | - | - |
|  |  |  | DB11677 | Triheptanoin | 1 | 0.99 | 1 | 0.9 |
| **58** | SW100 | 9-Hexadecen-1-ol, (Z)- | DB06894 | 1-Dodecanol | 0.95 | - | - | - |
| **59** | SW101 | 9,12-Octadecadienoic acid, methyl ester, (E,E)- | DB01631 | Methyl nonanoate | 0.95 | - | - | - |
| **60** | SW102 | Cis-13-Octadecenoic acid, methyl ester | DB01631 | Methyl nonanoate | 0.95 | - | - | - |
| **61** | SW103 | trans-13-Octadecenoic acid, methyl ester | DB01631 | Methyl nonanoate | 0.95 | - | - | - |
| **62** | SW104 | Phytol | DB02509 | Farnesol | 1 | - | - | - |
| **63** | SW105 | 10,13-Eicosadienoic acid, methyl ester | DB01631 | Methyl nonanoate | 0.95 | - | - | - |
| **64** | SW106 | Hexadecanoic acid, 2-hydroxy-1- | DB07416 | (2S)-2-(BUTYRYLOXY)-3-HYDROXYPROPYL NONANOATE | 0.88 | - | - | - |
| **65** | SW107 | (22S)-21-Acetoxy-6α,11β-dihydroxy-16α,17α-propylmethylenedioxypregna-1,4-diene-3,20-dione | DB00288 | Amcinonide | 0.89 | - | - | - |
|  |  |  | DB00511 | Acetyldigitoxin | 0.89 | - | - | - |
|  |  |  | DB00602 | Ivermectin | 0.86 | - | - | - |
|  |  |  | DB00846 | Flurandrenolide | 0.86 | - | - | - |
|  |  |  | DB01130 | Prednicarbate | 0.85 | - | - | - |
|  |  |  | DB01222 | Budesonide | 0.96 | - | - | - |
|  |  |  | DB01260 | Desonide | 0.92 | - | - | - |
|  |  |  | DB01380 | Cortisone acetate | 0.86 | - | - | - |
|  |  |  | DB01396 | Digitoxin | 0.85 | - | - | - |
|  |  |  | DB01410 | Ciclesonide | 0.92 | - | - | - |
|  |  |  | DB02169 | 9,10-Deepithio-9,10-Didehydroacanthifolicin | 0.85 | - | - | - |
|  |  |  | DB12499 | Clascoterone Methylprednisone | 0.86 | - | - | - |
|  |  |  | DB14538 | Hydrocortisone aceponate | 0.9 | - | - | - |
|  |  |  | DB14539 | Hydrocortisone acetate | 0.88 | - | - | - |
|  |  |  | DB14540 | Hydrocortisone butyrate | 0.9 | - | - | - |
|  |  |  | DB14541 | Hydrocortisone cypionate | 0.86 | - | - | - |
|  |  |  | DB14543 | Hydrocortisone probutate | 0.9 | - | - | - |
|  |  |  | DB14544 | Hydrocortisone valerate | 0.9 | - | - | - |
|  |  |  | DB14545 | Hydrocortisone succinate | 0.86 | - | - | - |
|  |  |  | DB14633 | Prednisolone hemisuccinate | 0.86 | - | - | - |
|  |  |  | DB14644 | Methylprednisolone hemisuccinate | 0.88 | - | - | - |
|  |  |  | DB14646 | Prednisone acetate | 0.86 | - | - | - |
|  |  |  | DB15566 | Prednisolone acetate | 0.88 | - | - | - |
| **66** | SW108 | Stigmast-5-en-3-ol, oleate | DB01420 | Testosterone propionate | 0.92 | - | - | - |
|  |  |  | DB06789 | Testosterone succinate | 0.88 | - | - | - |
|  |  |  | DB08804 | Nandrolone decanoate | 0.89 | - | - | - |
|  |  |  | DB13602 | Promegestone | 0.86 | - | - | - |
|  |  |  | DB13943 | Testosterone cypionate | 0.87 | - | - | - |
|  |  |  | DB13944 | Testosterone enanthate | 0.92 | - | - | - |
|  |  |  | DB13946 | Testosterone undecanoate | 0.92 | - | - | - |
|  |  |  | DB14639 | Boldenone undecylenate | 0.85 | - | - | - |
|  |  |  | DB14678 | Norethindrone enanthate | 0.87 | - | - | - |
| **67** | SW109 | Cholesterol | DB00146 | Calcifediol | 0.87 | - | - | - |
|  |  |  | DB00153 | Ergocalciferol | 0.91 | - | - | - |
|  |  |  | DB00169 | Cholecalciferol | 0.94 | - | - | - |
|  |  |  | DB01070 | Dihydrotachysterol | 0.94 | - | - | - |
|  |  |  | DB01431 | Allylestrenol | 0.86 | - | - | - |
|  |  |  | DB01564 | Calusterone | 0.86 | - | - | - |
|  |  |  | DB01708 | Prasterone | 0.86 | - | - | - |
|  |  |  | DB02789 | Pregnenolone | 0.89 | - | - | - |
|  |  |  | DB03696 | Lanosterol | 0.97 | - | - | - |
|  |  |  | DB04540 | Cholesterol | 1 | - | 1 | 1 |
|  |  |  | DB06710 | Methyltestosterone | 0.86 | - | - | - |
|  |  |  | DB11429 | Mibolerone | 0.86 | - | - | - |
| **68** | SW112 | Octadecanoic acid, 10-oxo-, methyl ester | DB01631 | Methyl nonanoate | 0.86 | - | 0.87 | - |
