# Supplementary material for: Computational and experimental insights into the interaction of the seaweed-derived steroidal metabolite 11α-hydroxyprogesterone with the glucocorticoid receptor
Source: Comput Struct Biotechnol J. 2025 Dec 30;31:202–20. doi: 10.1016/j.csbj.2025.12.028 (PMC12809411; doi:10.1016/j.csbj.2025.12.028)
Supplement: Table S4 — Supplementary material [file mmc4.docx]

**Table S2. Seaweed-derived metabolites exhibiting moderate structural similarity** **(Tc = 0.70–0.84) to approved drugs.**

| **No.** | **Accession number** | **Metabolites** |
| --- | --- | --- |
| 1 | SW004 | Octahydrobenzo[b]pyran, 4a-acetoxy-5,5,8a-trimethyl- |
| 2 | SW006 | 5,6,6-Trimethyl-5-(3-oxobut-1-enyl)-1-oxaspiro[2.5]octan-4-one |
| 3 | SW010 | 9-Fluoro-17alpha-methylandrost-4-ene-3alpha,6beta,11beta,17beta-tetrol |
| 4 | SW015 | 2-Pentadecanone, 6,10,14-trimethyl- |
| 5 | SW020 | Phthalic acid, butyl dodecyl ester |
| 6 | SW034 | 9,12,15-Octadecatrienoic acid, 2,3-dihydroxypropyl ester, (Z,Z,Z)- |
| 7 | SW037 | Methyl 9,12-epithio-9,11-octadecanoate |
| 8 | SW039 | Oxiraneoctanoic acid, 3-octyl-, cis- |
| 9 | SW040 | 6-Methyl-11-propenyl-5-(toluene-4-sulfonyloxy)-12,13-dioxatricyclo[7.3.1.0(1,6)]tridecane-8-carboxylic acid, methyl ester |
| 10 | SW045 | B(9a)-Homo-19-norpregna-9(11),9a-dien-20-one, 3-(dimethylamino)-4,4,14-trimethyl-, (3beta,5alpha)- |
| 11 | SW046 | 7,8-Epoxylanostan-11-ol, 3-acetoxy- |
| 12 | SW047 | Bufa-20,22-dienolide, 3-(acetyloxy)-14,15-epoxy-16-hydroxy-, (3beta,5beta,15beta,16beta)- |
| 13 | SW049 | 1H-Cyclopropa[3,4]benz[1,2-e]azulene-4a,5,7b,9,9a(1aH)-pentol, 3-[(acetyloxy)methyl]-1b,4,5,7a,8,9-hexahydro-1,1,6,8-tetramethyl-, 5,9,9a-triacetate, [1aR-(1aalpha,1bbeta,4abeta,5beta,7aalpha,7balpha,8alpha,9beta,9aalpha)]- |
| 14 | SW056 | Strobilactone A |
| 15 | SW060 | Fenretinide |
| 16 | SW061 | Cumanin |
| 17 | SW062 | Dihydroxanthin |
| 18 | SW063 | Propiolic acid, 3-(1-hydroxy-2-isopropyl-5-methylcyclohexyl)-, ethyl ester |
| 19 | SW066 | 9-Hexadecenoic acid, eicosyl ester, (Z)- |
| 20 | SW068 | 1,2-Benzenedicarboxylic acid, butyl octyl ester |
| 21 | SW069 | Methyl 5,6-diacetyloxy-10-hydroxy-2,4b,7,7,10a,12a-hexamethyl-12-methylidene-1,4,8-trioxo-4a,5,6,6a,9,10,10b,11-octahydronaphtho[1,2-h]isochromene-2-carboxylate |
| 22 | SW070 | Linoleic acid ethyl ester |
| 23 | SW073 | N-Retinoyl DL-phenylalanine (all-trans) |
| 24 | SW076 | Perhydroindene-4-carboxylic acid, 6-acetoxy-2,3-epoxy-1,1-epoxymethyl-3a-hydroxy-5-isopropenyl-7a-methyl-7-oxo-, methyl ester |
| 25 | SW078 | Incensole oxide |
| 26 | SW079 | 4,4-Difluororetinol (all-trans) |
| 27 | SW081 | 7,8,12-Tri-O-acetyl ingol |
| 28 | SW082 | 3Beta-chloro-5alpha-cholestane-5,6beta-diol 6-acetate |
| 29 | SW083 | 10aH-2,12a-Methano-1H,4H-cyclopropa[5,6][1,3]dioxolo[2',3']cyclopenta[1',2':9,10]cyclodeca[1,2-d][1,3]dioxin-15-ol, 1a,2,7a,13,14,14a-hexahydro-1,1,6,6,9,9,11,13-octamethyl-, [1aR-(1aα,2α,7aα,7bS*,10aα,12aα,13α,14aα,15R*)]- |
| 30 | SW085 | Bufa-20,22-dienolide, 14,15-epoxy-3,16-dihydroxy-, (3beta,5beta,15beta,16beta)- |
| 31 | SW086 | 9,11,18-Trihydroxy-6,18-epoxypimara-5,8(14),15-trien-7-one, 2Ac derivative |
| 32 | SW093 | 2(4H)-Benzofuranone, 5,6,7,7a-tetrahydro-4,4,7a-trimethyl-, (7aR)- |
| 33 | SW094 | 1-(2-[3-(2-Acetyloxiran-2-yl)-1,1-dimethylpropyl]cycloprop-2-enyl)ethanone |
| 34 | SW095 | 4,6,10,10-Tetramethyl-5-oxatricyclo[4,4,0,0(1,4)]dec-2-en-7-ol |
| 35 | SW097 | Tetradecane, 2,6,10-trimethyl- |
| 36 | SW098 | Heptadecane |
| 37 | SW099 | 3-Hydroxy-1a,5-bis(hydroxymethyl)-5,6b-dimethyl-1,3,3a,4,6,6a-hexahydrocyclopropa[e]inden-2-one |
