# Supplementary material for: Computational and experimental insights into the interaction of the seaweed-derived steroidal metabolite 11α-hydroxyprogesterone with the glucocorticoid receptor
Source: Comput Struct Biotechnol J. 2025 Dec 30;31:202–20. doi: 10.1016/j.csbj.2025.12.028 (PMC12809411; doi:10.1016/j.csbj.2025.12.028)
Supplement: Table S5 — Supplementary material [file mmc5.docx]

**Table S3.** Thirty-two inflammation-associated proteins identified from DisGeNET and UniProt. Information includes UniProt accession, gene symbol, protein name, and functional annotation related to inflammatory pathways.

| **No.** | **UniProt** | **Protein Name** | **Gene** | **Gene Full Name** |
| --- | --- | --- | --- | --- |
| **1** | O14842 | Free fatty acid receptor 1 | FFAR1 | Free fatty acid receptor 1 |
| **2** | O60427 | Fatty acid desaturase 1 | FADS1 | Fatty acid desaturase 1 |
| **3** | O60488 | Long-chain-fatty-acid--CoA ligase 4 | ACSL4 | Acyl-CoA synthetase long chain family member 4 |
| **4** | O95864 | Fatty acid desaturase 2 | FADS2 | Fatty acid desaturase 2 |
| **5** | P02774 | Vitamin D-binding protein | GC | GC vitamin D binding protein |
| **6** | P03372 | Estrogen receptor alpha | ESR1 | Estrogen receptor 1 |
| **7** | P04054 | Phospholipase A2 | PLA2G1B | Phospholipase A2 group IB |
| **8** | P04083 | Annexin A1 | ANXA1 | Annexin A1 |
| **9** | P04150 | Glucocorticoid receptor | NR3C1 | Nuclear receptor subfamily 3 group C member 1 |
| **10** | P04798 | Cytochrome P450 1A1 | CYP1A1 | Cytochrome P450 family 1 subfamily A member 1 |
| **11** | P06401 | Progesterone receptor | PGR | Progesterone receptor |
| **12** | P08183 | ATP-dependent translocase ABCB1 | ABCB1 | ATP binding cassette subfamily B member 1 |
| **13** | P08235 | Mineralocorticoid receptor | NR3C2 | Nuclear receptor subfamily 3 group C member 2 |
| **14** | P09917 | Polyunsaturated fatty acid 5-lipoxygenase | ALOX5 | Arachidonate 5-lipoxygenase |
| **15** | P11511 | Aromatase | CYP19A1 | Cytochrome P450 family 19 subfamily A member 1 |
| **16** | P17516 | Aldo-keto reductase family 1 C4 | AKR1C4 | Aldo-keto reductase family 1 member C4 |
| **17** | P19793 | Retinoic acid receptor RXR-alpha | RXRA | Retinoid X receptor alpha |
| **18** | P23219 | Prostaglandin G/H synthase 1 | PTGS1/COX1 | Prostaglandin-endoperoxide synthase 1 |
| **19** | P25963 | NF-kappa-B inhibitor alpha | NFKBIA | NFKB inhibitor alpha |
| **20** | P28845 | Corticosteroid 11-beta-dehydrogenase 1 | HSD11B1 | Hydroxysteroid 11-beta dehydrogenase 1 |
| **21** | P33261 | Cytochrome P450 2C19 | CYP2C19 | Cytochrome P450 family 2 subfamily C member 19 |
| **22** | P33527 | Multidrug resistance-associated protein 1 | ABCC1 | ATP binding cassette subfamily C member 1 (ABCC1 blood group) |
| **23** | P35354 | Prostaglandin G/H synthase 2 | PTGS2/COX2 | Prostaglandin-endoperoxide synthase 2 |
| **24** | P37231 | Peroxisome proliferator-activated receptor gamma | PPARG | Peroxisome proliferator activated receptor gamma |
| **25** | P42330 | Aldo-keto reductase family 1 C3 | AKR1C3 | Aldo-keto reductase family 1 member C3 |
| **26** | Q02318 | Sterol 26-hydroxylase, mitochondrial | CYP27A1 | Cytochrome P450 family 27 subfamily A member 1 |
| **27** | Q03181 | Peroxisome proliferator-activated receptor delta | PPARD | Peroxisome proliferator activated receptor delta |
| **28** | Q07869 | Peroxisome proliferator-activated receptor alpha | PPARA | Peroxisome proliferator activated receptor alpha |
| **29** | Q13936 | Voltage-dependent L-type calcium channel subunit alpha-1C | CACNA1C | Calcium voltage-gated channel subunit alpha1 C |
| **30** | Q8NER1 | Transient receptor potential cation channel subfamily V member 1 | TRPV1 | Transient receptor potential cation channel subfamily V member 1 |
| **31** | Q96RI1 | Bile acid receptor | NR1H4 | Nuclear receptor subfamily 1 group H member 4 |
| **32** | Q9H015 | Solute carrier family 22 member 4 | SLC22A4 | Solute carrier family 22 member 4 |
