# Supplementary material for: Computational and experimental insights into the interaction of the seaweed-derived steroidal metabolite 11α-hydroxyprogesterone with the glucocorticoid receptor
Source: Comput Struct Biotechnol J. 2025 Dec 30;31:202–20. doi: 10.1016/j.csbj.2025.12.028 (PMC12809411; doi:10.1016/j.csbj.2025.12.028)
Supplement: Table S6 — Supplementary material [file mmc6.docx]

**Table S4.** Selected protein targets, structurally similar drugs, and seaweed-derived metabolites selected for molecular docking analysis based on network centrality, druggability, and structural similarity criteria.

| **No.** | **Uniprot** | **Protein** | **Type** | **Drug ID** | **Drug** | **Accession Number** |
| --- | --- | --- | --- | --- | --- | --- |
| 1 | P04150 | Glucocorticoid receptor | target | DB00180 | Flunisolide | SW048 |
|  |  |  |  | DB00253 | Medrysone | SW005 |
|  |  |  |  |  |  | SW052 |
|  |  |  |  | DB00288 | Amcinonide | SW048 |
|  |  |  |  |  |  | SW107 |
|  |  |  |  | DB00324 | Fluorometholone | SW010 |
|  |  |  |  | DB00591 | Fluocinolone acetonide | SW048 |
|  |  |  |  | DB00635 | Prednisone | SW088 |
|  |  |  |  | DB00741 | Hydrocortisone | SW088 |
|  |  |  |  | DB00764 | Mometasone | SW010 |
|  |  |  |  | DB00846 | Flurandrenolide | SW048 |
|  |  |  |  |  |  | SW107 |
|  |  |  |  | DB00860 | Prednisolone | SW088 |
|  |  |  |  | DB00896 | Rimexolone | SW052 |
|  |  |  |  | DB00959 | Methylprednisolone | SW048 |
|  |  |  |  |  |  | SW088 |
|  |  |  |  | DB01130 | Prednicarbate | SW107 |
|  |  |  |  | DB01222 | Budesonide | SW048 |
|  |  |  |  |  |  | SW107 |
|  |  |  |  | DB01260 | Desonide | SW048 |
|  |  |  |  |  |  | SW107 |
|  |  |  |  | DB01380 | Cortisone acetate | SW107 |
|  |  |  |  | DB01410 | Ciclesonide | SW048 |
|  |  |  |  |  |  | SW107 |
|  |  |  |  | DB14538 | Hydrocortisone aceponate | SW048 |
|  |  |  |  |  |  | SW107 |
|  |  |  |  | DB14539 | Hydrocortisone acetate | SW048 |
|  |  |  |  |  |  | SW107 |
|  |  |  |  | DB14540 | Hydrocortisone butyrate | SW048 |
|  |  |  |  |  |  | SW107 |
|  |  |  |  | DB14541 | Hydrocortisone cypionate | SW107 |
|  |  |  |  | DB14543 | Hydrocortisone probutate | SW048 |
|  |  |  |  |  |  | SW107 |
|  |  |  |  | DB14544 | Hydrocortisone valerate | SW048 |
|  |  |  |  |  |  | SW107 |
|  |  |  |  | DB15566 | Prednisolone acetate | SW048 |
| 2 | P06401 | Progesterone receptor | target | DB00764 | Mometasone | SW010 |
| 3 | P28845 | Corticosteroid 11-beta-dehydrogenase isozyme 1 | enzyme | DB00635 | Prednisone | SW088 |
|  |  |  |  | DB00741 | Hydrocortisone | SW088 |
|  |  |  |  | DB00959 | Methylprednisolone | SW088 |
| 4 | P33261 | Cytochrome P450 2C19 | enzyme | DB00635 | Prednisone | SW088 |
|  |  |  |  | DB00741 | Hydrocortisone | SW088 |
|  |  |  |  | DB00959 | Methylprednisolone | SW088 |
|  |  |  |  | DB01222 | Budesonide | SW107 |
| 5 | P42330 | Aldo-keto reductase family 1 C3 | enzyme | DB00959 | Methylprednisolone | SW088 |

Note: SW005: Propanoic acid, 2-(3-acetoxy-4,4,14-trimethylandrost-8-en-17-yl)-; SW010: 9α-Fluoro-17α-methyl-4-androsten-3α, 6β,11β,17β-tetra-ol; SW048: (22S)-6α,11β,21-Trihydroxy-16α,17α-propylmethylenedioxypregna-1,4-diene-3,20-dione; SW052: 11α-hydroxyprogesterone; SW088: Olean-12-ene-3,15,16,21,22,28-hexol, (3β,15α,16α,21β,22α)-; SW107: (22S)-21-Acetoxy-6α,11β-dihydroxy-16α,17α-propylmethylenedioxypregna-1,4-diene-3,20-dione
