# Supplementary material for: Computational and experimental insights into the interaction of the seaweed-derived steroidal metabolite 11α-hydroxyprogesterone with the glucocorticoid receptor
Source: Comput Struct Biotechnol J. 2025 Dec 30;31:202–20. doi: 10.1016/j.csbj.2025.12.028 (PMC12809411; doi:10.1016/j.csbj.2025.12.028)
Supplement: Table S7 — Supplementary material [file mmc7.docx]

**Tables S5.** Summarize the binding affinities of control ligands across ten docking replicates, as well as the binding energies of seaweed-derived compounds and control ligands against Glucocorticoid receptor (P04150)

| **Ligands** | **Binding affinity (kcal/mol)** | | | | | | | | | | |
| --- | --- | --- | --- | --- | --- | --- | --- | --- | --- | --- | --- |
|  | **rep1** | **rep2** | **rep3** | **rep4** | **rep5** | **rep6** | **rep7** | **rep8** | **rep9** | **rep10** | **mode of the best-pose binding energies** |
| **Control ligands (Fingerprint ligand)** | | | | | | | | | | | |
| DB00180 | -8.8 | -8.8 | -8.8 | -8.8 | -8.8 | -8.8 | -8.8 | -8.8 | -8.8 | -8.8 | -8.8 |
| DB00253 | -11.6 | -11.6 | -11.7 | -11.6 | -11.6 | -11.6 | -11.6 | -11.6 | -11.6 | -11.6 | -11.6 |
| DB00288 | -6.7 | -6.7 | -6.7 | -6.7 | -6.7 | -6.7 | -6.7 | -6.7 | -6.7 | -6.7 | -6.7 |
| DB00324 | -11 | -11 | -11 | -11 | -11 | -11 | -11 | -11 | -11 | -11 | -11 |
| DB00591 | -8.9 | -8.9 | -8.9 | -8.9 | -8.9 | -8.9 | -8.9 | -8.9 | -8.9 | -8.9 | -8.9 |
| DB00635 | -11.5 | -11.5 | -11.5 | -11.5 | -11.5 | -11.5 | -11.5 | -11.5 | -11.5 | -11.5 | -11.5 |
| DB00764 | -11.5 | -11.5 | -11.5 | -11.5 | -11.5 | -11.5 | -11.5 | -11.5 | -11.5 | -11.5 | -11.5 |
| DB00846 | -9.7 | -9.6 | -9.6 | -9.6 | -9.6 | -9.6 | -9.6 | -9.6 | -9.6 | -9.6 | -9.6 |
| DB00860 | -11.2 | -11.2 | -11.2 | -11.2 | -11.2 | -11.2 | -11.2 | -11.2 | -11.2 | -11.2 | -11.2 |
| DB00896 | -11.8 | -11.8 | -11.8 | -11.8 | -11.8 | -11.8 | -11.8 | -11.8 | -11.8 | -11.8 | -11.8 |
| DB00959 | -10.5 | -10.5 | -10.5 | -10.5 | -10.5 | -10.5 | -10.5 | -10.5 | -10.5 | -10.5 | -10.5 |
| DB01130 | -7.7 | -7.7 | -7.6 | -7.7 | -7.7 | -7.7 | -7.7 | -7.6 | -7.7 | -7.7 | -7.7 |
| DB01222 | -10.1 | -10.1 | -10.1 | -10.1 | -10.1 | -10.1 | -10.1 | -10.1 | -10.1 | -10.1 | -10.1 |
| DB01260 | -8.7 | -8.7 | -8.7 | -8.7 | -8.7 | -8.7 | -8.7 | -8.7 | -8.7 | -8.7 | -8.7 |
| DB01380 | -9.4 | -9.4 | -9.4 | -9.5 | -9.4 | -9.4 | -9.5 | -9.4 | -9.4 | -9.4 | -9.4 |
| DB01410 | -4.2 | -3.9 | -4.2 | -4.2 | -4.2 | -4.2 | -4.2 | -3.9 | -4.2 | -4.2 | -4.2 |
| DB14538 | -8.9 | -9 | -8.9 | -8.9 | -8.9 | -8.9 | -8.9 | -8.9 | -8.9 | -9 | -8.9 |
| DB14539 | -9.6 | -9.6 | -9.6 | -9.6 | -9.6 | -9.7 | -9.7 | -9.7 | -9.6 | -9.7 | -9.6 |
| DB14540 | -10.8 | -10.8 | -10.8 | -10.8 | -10.8 | -10.8 | -10.8 | -10.8 | -10.8 | -10.8 | -10.8 |
| DB14541 | -8.5 | -8.6 | -8.6 | -8.6 | -8.6 | -8.5 | -8.6 | -8.5 | -8.6 | -8.5 | -8.6 |
| DB14543 | -7.8 | -7.7 | -7.7 | -7.9 | -7.8 | -7.7 | -7.8 | -7.8 | -7.8 | -7.8 | -7.8 |
| DB14544 | -10.7 | -10.7 | -10.7 | -10.7 | -10.7 | -10.7 | -10.7 | -10.7 | -10.7 | -10.7 | -10.7 |
| DB15566 | -9.7 | -9.7 | -9.7 | -9.7 | -9.7 | -9.7 | -9.7 | -9.7 | -9.7 | -9.7 | -9.7 |
| **Control ligands (Co-crystallized ligand)** | | | | | | | | | | | |
| DB00741 | -11.4 | -11.4 | -11.4 | -11.4 | -11.4 | -11.4 | -11.4 | -11.5 | -11.4 | -11.4 | -11.4 |
| **Control ligands** | | | | | | | | | | | |
| DB01234 | -11.1 | -11.1 | -11.1 | -11.1 | -11.1 | -11.1 | -11.1 | -11.1 | -11.1 | -11.1 | -11.1 |
| **Seaweed-derived metabolite** | | | | | | | | | | | |
| SW005 | -4.1 | -4.1 | -4.1 | -4.1 | -4.1 | -4.1 | -4.1 | -4.1 | -4.1 | -4.1 | -4.1 |
| SW010 | -10.5 | -10.5 | -10.5 | -10.5 | -10.5 | -10.5 | -10.5 | -10.5 | -10.5 | -10.5 | -10.5 |
| SW048 | -5.4 | -5.4 | -5.4 | -5.4 | -5.4 | -5.4 | -5.4 | -5.4 | -5.4 | -5.4 | -5.4 |
| SW052 | -11.6 | -11.6 | -11.6 | -11.6 | -11.6 | -11.6 | -11.6 | -11.6 | -11.6 | -11.6 | -11.6 |
| SW088 | 10.7 | 10.7 | 10.7 | 10.8 | 10.8 | 10.8 | 10.7 | 10.7 | 10.8 | 10.8 | 10.8 |
| SW107 | -5 | -5 | -5 | -5 | -5 | -5 | -5 | -5 | -5 | -5 | -5 |
