# Supplementary material for: Computational and experimental insights into the interaction of the seaweed-derived steroidal metabolite 11α-hydroxyprogesterone with the glucocorticoid receptor
Source: Comput Struct Biotechnol J. 2025 Dec 30;31:202–20. doi: 10.1016/j.csbj.2025.12.028 (PMC12809411; doi:10.1016/j.csbj.2025.12.028)
Supplement: Table S9 — Supplementary material [file mmc9.docx]

**Tables S7.** Summarize the binding affinities of control ligands across ten docking replicates, as well as the binding energies of seaweed-derived compounds and control ligands against the Corticosteroid 11-beta-dehydrogenase isozyme 1 (P28845)

| **Ligands** | **Binding affinity (kcal/mol)** | | | | | | | | | | |
| --- | --- | --- | --- | --- | --- | --- | --- | --- | --- | --- | --- |
|  | **rep1** | **rep2** | **rep3** | **rep4** | **rep5** | **rep6** | **rep7** | **rep8** | **rep9** | **rep10** | **mode of the best-pose binding energies** |
| **Control ligands (Fingerprint ligand)** | | | | | | | | | | | |
| DB00635 | -9.6 | -9.6 | -9.6 | -9.6 | -9.6 | -9.6 | -9.6 | -9.6 | -9.6 | -9.6 | -9.6 |
| DB00741 | -9.1 | -9.1 | -9.1 | -9.1 | -9.1 | -9.1 | -9.1 | -9.1 | -9.1 | -9.1 | -9.1 |
| DB00959 | -9.1 | -9.4 | -9.1 | -9.1 | -9.1 | -9.1 | -9.1 | -9.1 | -9.1 | -9.1 | -9.1 |
| **Control ligands (Co-crystallized ligand)** | | | | | | | | | | | |
| DB08277 | -9.8 | -9.8 | -9.8 | -9.8 | -9.8 | -9.8 | -9.8 | -9.8 | -9.8 | -9.8 | -9.8 |
| **Seaweed-derived metabolite** | | | | | | | | | | | |
| SW048 | -9.9 | -9.9 | -9.9 | -9.9 | -9.9 | -9.9 | -9.9 | -9.9 | -9.9 | -9.9 | -9.9 |
| SW088 | -9.1 | -9.1 | -9.1 | -9.1 | -9.1 | -9.1 | -9.1 | -9.1 | -9.1 | -9.1 | -9.1 |
